# Polymer Solar Cells—Interfacial Processes Related to Performance Issues

**DOI:** 10.3389/fchem.2019.00061

**Published:** 2019-02-12

**Authors:** Abhay Gusain, Roberto M. Faria, Paulo B. Miranda

**Affiliations:** Instituto de Física de São Carlos, Universidade de São Paulo, São Carlos, Brazil

**Keywords:** polymer solar cells, device performance, interfaces, energy barriers, interfacial dipoles

## Abstract

Harnessing solar energy with solar cells based on organic materials (in particular polymeric solar cells) is an attractive alternative to silicon-based solar cells due to the advantages of lower weight, flexibility, lower manufacturing costs, easier integration with other products, low environmental impact during manufacturing and operations and short energy payback times. However, even with the latest efficiencies reported up to 17%, the reproducibility of these efficiencies is not up to par, with a significant variation in the efficiencies reported across the literature. Since these devices are based on ultrathin multilayer organic films, interfaces play a major role in their operation and performance. This review gives a concise account of the major interfacial issues that are responsible for influencing the device performance, with emphasis on their physical mechanisms. After an introduction to the basic principles of polymeric solar cells, it briefly discusses charge generation and recombination occurring at the donor-acceptor bulk heterojunction interface. It then discusses interfacial morphology for the active layer and how it affects the performance and stability of these devices. Next, the formation of injection and extraction barriers and their role in the device performance is discussed. Finally, it addresses the most common approaches to change these barriers for improving the solar cell efficiency, including the use of interface dipoles. These issues are interrelated to each other and give a clear and concise understanding of the problem of the underperformance due to interfacial phenomena occurring within the device. This review not only discusses some of the implemented approaches that have been adopted in order to address these problems, but also highlights interfacial issues that are yet to be fully understood in organic solar cells.

**Graphical Abstract:**
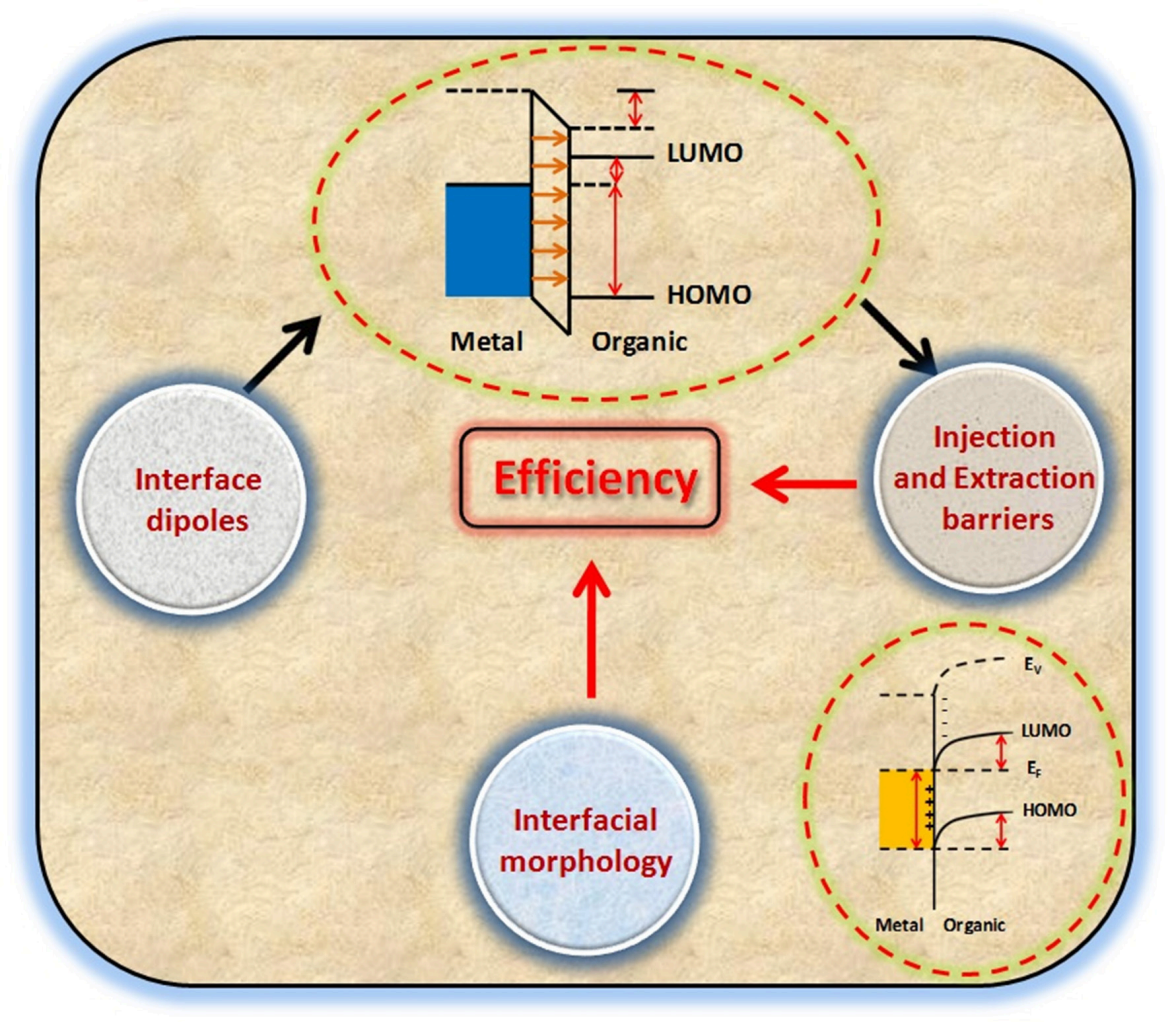
Factors related to interfaces that affect the overall device performance.

## Introduction

Polymer-based solar cells have been the subject of more focused and continuous research since last decade, which saw a drastic increase of their power conversion efficiencies from 6% up to 17% within less than a decade, as shown in the Table 1 (Dam et al., 1999; Winder and Sariciftci, 2004; Mühlbacher et al., 2006; Vanlaeke et al., 2006; Hou et al., 2008; Jørgensen et al., 2008; Park et al., 2009; Norrman et al., 2010; Tsai et al., 2010; Chu et al., 2011; Albrecht et al., 2012; He et al., 2012, 2015; Li et al., 2012, 2018; Lu et al., 2013; You et al., 2013; Zhang et al., 2013; Zhou et al., 2013, 2015; Chi et al., 2014; Liu et al., 2014b; Liu S. et al., 2015; Meng et al., 2018). Such remarkable achievement has only been possible with the introduction of new materials, including the low bandgap polymers and new fullerene derivatives (Spanggaard and Krebs, 2004; Brabec et al., 2005; Bundgaard and Krebs, 2007; Rand et al., 2007; Kroon et al., 2008) and other acceptor molecules (Li et al., 2018; Meng et al., 2018), improving the properties of the existing materials, such as the solubility and bandgap of fullerenes (Spanggaard and Krebs, 2004; Krebs, 2005; Shaheen et al., 2005), advances in device architecture (Coakley and McGehee, 2004; Janssen et al., 2005), addition of new buffer layers in the conventional architecture and adoption of novel approaches for thermal and solvent annealing (Coakley et al., 2005; Mayer et al., 2007), among others. Indeed, the problem of lower efficiencies of polymer solar cells with respect to other organic or hybrid approaches [such as the Grätzel cell (Gratzel, 2005) and organic-inorganic perovskite solar cells (Hu et al., 2017)] has been attributed to factors like device architecture, materials used for fabrication of the solar cells and their properties (molecular weight of the donor polymer, purity of the materials, energy level alignments and band gap) (Bundgaard and Krebs, 2007; Rand et al., 2007; Tress et al., 2011), the processing parameters and conditions during the fabrication of the solar cells, such as spin-coating conditions, solvent and additives used (Wu et al., 2011), thermal and solvent annealing treatment and their duration (Coakley et al., 2005; Mayer et al., 2007), which determine the thickness and morphology of the various organic layers and interfaces (Matturro et al., 1986; Lögdlund and Brédas, 1994; de Jong et al., 2000; Norrman et al., 2006; Tress et al., 2011; Wu et al., 2011; Gusain et al., 2013). Apart from their efficiency, other relevant aspects of the device performance are the stability and the degradation of the polymer solar cells during operation (Norrman et al., 2006). These have also been attributed to material properties which make them prone to undergo structural changes after reaction with the ambient oxygen and moisture when they are exposed to them (Matturro et al., 1986; Lögdlund and Brédas, 1994; Dam et al., 1999; de Jong et al., 2000; Norrman et al., 2006, 2010; Jørgensen et al., 2008).

**Table 1:** Summary of recently reported efficiencies of different polymer BHJ solar cells.

**S. No**.	**Donor polymer/acceptor material**	**Architecture**	**Bandgap (eV)**	**Efficiency (%)**	**Year**	**References**
1.	PTPTB:PC_61_BM	Single junction	1.7–2.1	1	2004	Winder and Sariciftci, 2004
2.	PEOPT:PC_61_BM	Single junction	1.75	0.02	2004	Winder and Sariciftci, 2004
3.	PFDTBT:PC_61_BM	Single junction	1.9	2	2004	Winder and Sariciftci, 2004
4.	P3HT:PC_61_BM	Single junction	2.1	2.8	2006	Vanlaeke et al., 2006
5.	PCPDTBT:PC_71_BM	Single junction	1.70	3.2	2006	Mühlbacher et al., 2006
6.	PCPDTBT:PC_70_BM	Single junction	1.70	5.1	2008	Hou et al., 2008
7.	PCDTBT:PC_70_BM	Single junction	1.8	6.1	2009	Park et al., 2009
8.	P3HT:PC_61_BM	Single junction	2.1	4.4	2010	Tsai et al., 2010
9.	PCDTBT:PC_70_BM	Single junction	1.8	7.1	2011	Chu et al., 2011
10.	P3HT:PC_61_BM	Single junction	2.1	3.37	2012	Albrecht et al., 2012
11.	P3HT:PC_61_BM	Single junction	2.1	3.68	2012	Li et al., 2012
12.	P3HT:PC_61_BM	Single junction	2.1	3.9	2012	Albrecht et al., 2012
13.	PCPDTBT:PC_70_BM	Single junction	1.70	6.16	2012	Albrecht et al., 2012
14.	P3HT:PC_61_BM	Single junction	2.1	4.24	2013	Zhou et al., 2013
15.	PTB7:PC_70_BM	Single junction	1.6	7.9	2013	Zhou et al., 2013
16.	PBDTP-DTBT:PC_71_BM	Single junction	1.70	8.07	2013	Zhang et al., 2013
17.	PTB7:PC_70_BM	Single junction	1.6	8.67	2013	Lu et al., 2013
18.	P3HT: ICBA/PDTP-DFBT:PC_61_BM	Tandem junction	1.24	10.6	2013	You et al., 2013
19.	P3HT:PC_61_BM	Single junction	2.1	4.24	2014	Chi et al., 2014
20.	PCDTBT:PC_70_BM	Single junction	1.8	7.20	2014	Liu et al., 2014b
21.	PDVT-10/PBDTTT-EFT:PC_71_BM	Single junction	1.84	10.08	2015	Liu S. et al., 2015
22.	PTB7-Th/ZnO/CPEs:PC_71_BM	Tandem junction	1.6	11.3	2015	Zhou et al., 2015
23.	PBDB-TF:IT-4F	Single junction	1.89	13.7	2018	Li et al., 2018
24.	PFN-Br/PBDB-T:FM/PTB7-Th:O6T-4F:PC_71_BM	Tandem junction	1.25	17.3	2018	Meng et al., 2018

***PTPTB**—poly-N-dodecyl-2,5,-bis(2'-thienyl)pyrrole, 2,1,3-benzothiadiazole; **PEOPT**—poly(3-(4'-(1″,4″,7″-trioxaoctyl)phenyl)thiophene); **PFDTBT**—poly{[2,7-(9-(20-ethylhexyl)-9-hexylfluorene])-alt-[5,50-(40,70-di-2-thienyl-20,10,30-benzothiadiazole)]}; **P3HT**—(poly(3-hexyl)thiophene); **PCPDTBT**—poly[2,6-(4,4-bis(2-ethylhexyl)-4H-cyclopenta [2,1-b;3,4-b′]dithiophene)-alt-4,7(2,1,3-benzothiadiazole)]; **PCDTBT**—poly[N-9′-heptadecanyl-2,7-carbazole-alt-5,5-(4′,7′-di-2-thienyl-2′,1′,3′-benzothiadiazole)]; **PBDTP-DTBT**—poly{4,8-bis(4-(2-ethylhexyl)-phenyl)- benzo[1,2-b:4,5-b′]dithiophene-alt-[4,7-di(4-(2-ethylhexyl)-2- thienyl)-2,1,3-benzothiadiazole)-5, 5′-diyl]}; **ICBA**—indene-C_60_ bisadduct; **PDTP-DFBT**—poly[2,7-(5,5-bis-(3,7-dimethyl octyl)-5H-dithieno[3,2-b:2′,3′-d]pyran)-alt-4,7-(5,6-difluoro-2,1,3-benzothiadiazole)]; **PDVT-10**—poly[2,5-bis(alkyl)pyrrolo[3,4-c]pyrrole-1,4(2H,5H)-dione-alt-5,5'-i(thiophen-2-yl)-2,2'-(E)-2-(2-(thiophen2-yl)vinyl)thiophene]; **PBDTTT-EFT**—poly(2-ethylhexyl 6-(4,8-bis(5-(2-ethylhexyl)thiophen-2-yl)benzo[1,2-b:4,5-b′]dithiophen-2-yl)-3-fluorothieno[3,4-b]thiophene-2-carboxylate); **PTB7-Th**—poly[4,8-bis(5-(2-ethylhexyl)thiophen-2-yl)benzo[1,2-b:4,5-b′] dithiophene- co−3-fluorothieno[3,4-b]thiophene-2-carboxylate]; **PC**_**61**_**BM**—([6,6]-phenyl C_61_ butyric methyl ester); **PFN-Br**—poly[(9,9-bis{30-[N,N-dimethyl]-N-ethylammonium]propyl}-2,7-fluorene)-alt-1,4 phenylene]dibromide; **PBDB-TF**—poly{[4,8-bis[5-(2- ethylhexyl)-4-fluoro-2-thienyl]benzo[1,2- b:4,5-b′]dithiophene2,6-diyl]-alt-[2,5-thiophenediyl[5,7-bis(2-ethylhexyl)-4,8-dioxo4H,8H-benzo[1,2-c:4,5-c′]dithiophene-1,3-diyl]]}; **IT-4F**—fluorinated ITIC or 4F-ITIC; **ITIC**—(3,9-bis(2-methylene-(3-(1,1-dicyanomethylene)-indanone))-5,5,11,11-tetrakis(4-hexylphenyl)-dithieno[2,3-d:2',3'-d']-s-indaceno[1,2-b:5,6-b']dithiophene)); **PBDB-T**—poly[(2,6-(4,8-bis(5-(2-ethylhexyl)thiophen-2-yl)-benzo[1,2-b:4,5-b′]dithiophene))-alt-(5,5-(1′,3′-di-2-thienyl-5′,7′-bis(2-ethylhexyl)benzo[1′,2′-c:4′,5′-c′]dithiophene-4,8-dione))]; **FM**—methyl substituted F-H (2,9-bis(2-methylene(3-(1,1-dicyanomethylene)indanone))7,12-dihydro-4,4,7,7,12,12-hexaoctyl-4H-cyclopenta[2″,1″:5,6;3″,4″:5′,6′]diindeno[1,2-b:1′,2′-b′]dithiophene); **O6T-4F** or **COi8DFIC**—carbon-oxygen-bridged i8 difluoro-substituted 1,1-dicyanomethylene-3-indanone; **CPEs**—conjugated polyelectrolytes*.

Even though numerous approaches have been adopted to understand the factors behind this underperformance and methods have been developed to address them during the timeline of research on polymer solar cells, more recently it is becoming increasingly apparent that interfaces play a crucial role on the device performance and stability (Matturro et al., 1986; Lögdlund and Brédas, 1994; Dam et al., 1999; de Jong et al., 2000; Brabec et al., 2001b, 2005; Coakley and McGehee, 2004; Hoppe and Sariciftci, 2004; Spanggaard and Krebs, 2004; Winder and Sariciftci, 2004; Coakley et al., 2005; Janssen et al., 2005; Krebs, 2005; Shaheen et al., 2005; Mühlbacher et al., 2006; Norrman et al., 2006, 2010; Vanlaeke et al., 2006; Bundgaard and Krebs, 2007; Günes et al., 2007; Lloyd et al., 2007; Mayer et al., 2007; Rand et al., 2007; Hou et al., 2008; Jørgensen et al., 2008; Kroon et al., 2008; Thompson and Fréchet, 2008; Park et al., 2009; Tsai et al., 2010; Chu et al., 2011; Tress et al., 2011; Wu et al., 2011; Albrecht et al., 2012; He et al., 2012, 2015; Li et al., 2012; Gusain et al., 2013; Lu et al., 2013; You et al., 2013; Zhang et al., 2013; Zhou et al., 2013, 2015; Chi et al., 2014; Liu et al., 2014b; Liu S. et al., 2015). Indeed this should be expected, since these devices are based on ultrathin multilayer organic films, and charge has to transfer across many interfaces involving both organic and inorganic materials of widely varying properties. Therefore, the presentation of a review exclusively focusing on interfacial issues in polymer-based solar cells becomes important. In fact, several of these issues will also be relevant to other organic or hybrid thin film cells as well. This review starts off with a description of the basic device physics and architectures used in polymeric solar cells, followed by an account of the most relevant physical phenomena occurring at interfaces that affect solar cell performance and stability, described at the molecular level and grouped in four sections: (i) charge generation and recombination at donor-acceptor interfaces; (ii) the issue of interfacial morphology and its impact on the device performance and stability; (iii) formation of injection and extraction barriers and their role in the cell electrical performance; (iv) approaches used to control barriers, including the use of interface dipoles.

## Basic Device Physics and Architectures

**Figure 1:**
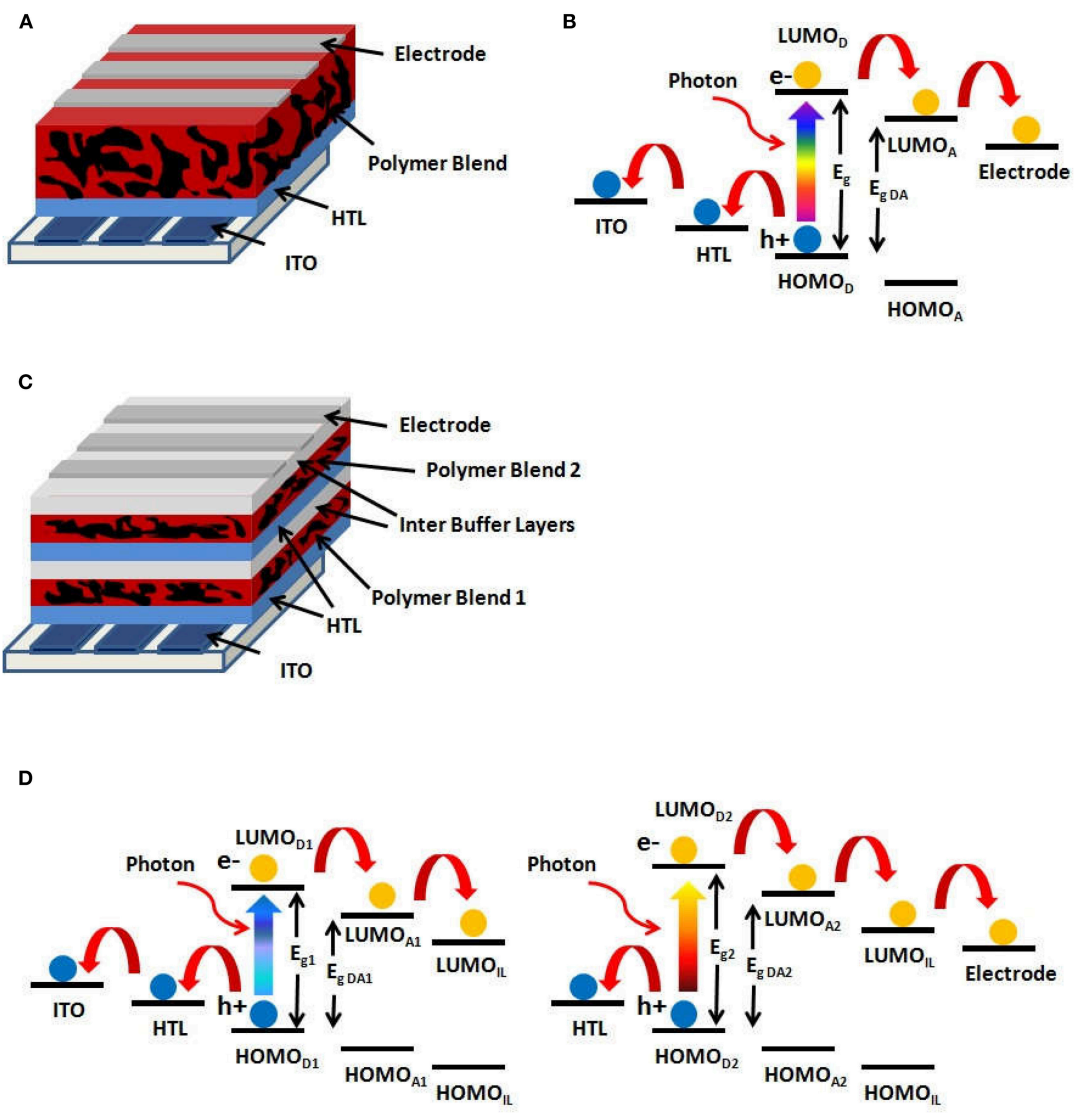
**(A)** Single junction BHJ architecture. **(B)** Energy level diagram for a single junction BHJ cell. **(C)** Tandem junction BHJ architecture. **(D)** Energy level diagrams for a tandem junction BHJ cell.

The mechanism of direct conversion of the energy of an absorbed photon into electrical energy is only possible in a photovoltaic device whose basic principle occurs in a semiconductor element exhibiting an electronic gap equal to or smaller than the energy of the absorbed photon (*h*ν). The immediate consequence of this absorption is the generation of an electron-hole pair; then, under the action of an internal field generated by the electrodes work function difference, the free charge carriers are conducted to the respective electrodes where they are captured. In a bulk heterojunction solar cell (BHJ), the absorbing component is an ultrathin active layer, usually comprised of a conjugated polymer mixed with an electronegative molecule, forming a nanostructured blend. A typical single junction organic BHJ device is shown in Figure 1A and consists of layers of different materials, such as a transparent bottom electrode of indium–tin oxide (ITO), a hole transport layer (HTL) like PEDOT:PSS (poly(3,4-ethylenedioxythiophene):polystyrene sulfonate), the donor polymer/acceptor molecule blend as the active layer and a top electrode layer (usually metallic). In contrast, a tandem junction BHJ cell, as shown in Figure 1C, consists of multiple stacks of the single junction BHJ, each having different combinations of donor/acceptor active layers, with interlayers (IL) serving to match the charge transport across both cells.

The energy level diagram for a BHJ solar cell is shown in Figure 1B. As the photon of the incident light is absorbed by the donor polymer in the active layer, the electron of the Highest Occupied Molecular Orbital (HOMO) of the donor polymer is excited to its Lowest Unoccupied Molecular Orbital (LUMO), creating a Coulombically bound electron-hole pair known as exciton. Such exciton diffuses within the donor polymer until it reaches the donor/acceptor interface, where it is dissociated by the energy favorable electron transfer from the LUMO_D_ of the donor polymer to the LUMO_A_ of the fullerene acceptor. In this process, the donor-acceptor LUMO energy difference for the electron is lost to vibrations (heat). Subsequently, the electron is transported through the acceptor phase and across the acceptor/metal electrode interface, while the hole left in the HOMO_D_ of the donor polymer is transported through it, collected at the HTL and transported across the HTL/ITO electrode interface. At each interface, the energy offset is lost to heat as the charge transfers across the interface. Therefore, minimizing these offsets has a direct impact in increasing the open-circuit voltage of the solar cell, and therefore its power conversion efficiency. A similar cascade of charge transfer occurs if light is absorbed in the acceptor molecule, except that the first step (exciton dissociation) is now due to a favorable hole transfer from the HOMO_A_ of the acceptor to the HOMO_D_ of the donor.

**Figure 2:**
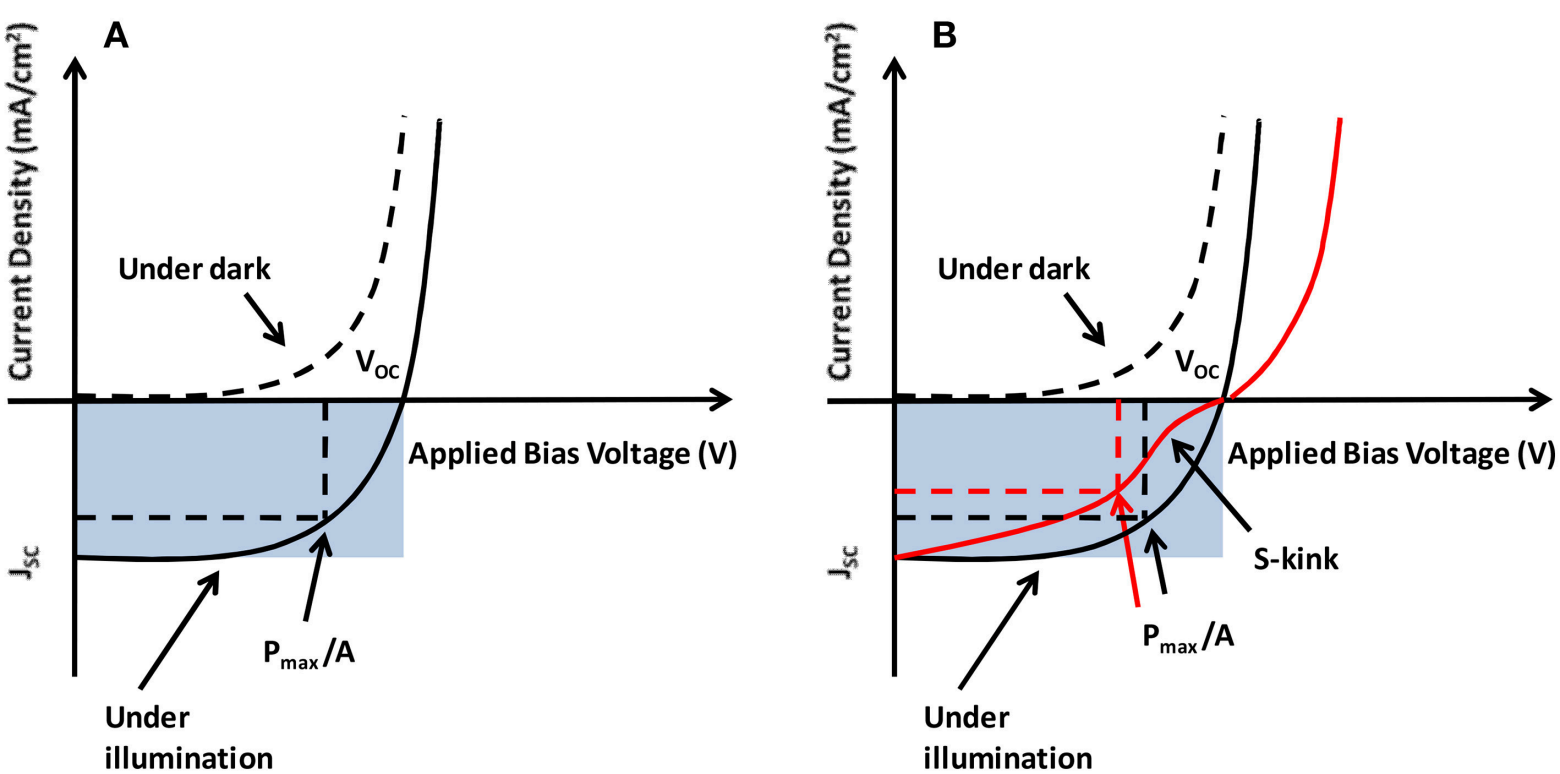
**(A)** The current-voltage characteristics of a solar cell and the photovoltaic parameters. **(B)** J-V curve with and without an S-kink.

The fundamental electrical characterization of a photovoltaic diode is carried out by obtaining curves of electric current density vs. an external voltage (bias), the so-called J-V curves. A typical J-V curve for a solar cell is shown in Figure 2, in the dark (dashed line) and under illumination (solid line). The photocurrent density *J*_*ph*_ is a subtraction between the two J-V curves (illuminated and dark), which are described by the Equations (1, 2), where *J*_o_ is the reverse dark current, *V* is the bias voltage, *n* is the diode quality factor, *k* is the Boltzmann constant and *T* is the temperature (Sze and Kwok Ng, 2007).

(1) $$\begin{array}{r} J\left(V\right)=J_{o}\left[exp\left(\frac{eV}{nKT}\right)-1\right]\;\left(Shockley^{\prime }s\;equation\right) \end{array}$$

(2) $$\begin{array}{r} J\left(V\right)=J_{o}\left[exp\left(\frac{eV}{nKT}\right)-1\right]+J_{ph}\;\left(under\;illumination\right) \end{array}$$

The parameters that characterize solar cells in general are shown in Figure 2: the short-circuit current density (*J*_*sc*_), the open-circuit voltage (*V*_*oc*_) and the maximum operating power (*P*_*max*_), which determine the fill factor (FF). The open circuit voltage is defined as the maximum voltage that is obtained when no current is generated by the solar cell. Similarly, the short circuit current density is the maximum current density that is obtained when there is no voltage across the solar cell. The fill factor is defined as the ratio of the maximum operating power (*P*_*max*_) to the maximum extractable power from an ideal solar cell, which would be the product of the device area *A, V*_*oc*_, and *J*_*sc*_. Thus, the power conversion efficiency is the ratio of the maximum operating power *P*_*max*_ to the input power of the incident light on the solar cell. Therefore, under an incident light intensity *I*_*in*_, the *FF* and the power conversion efficiency (η) are given by Equations (3, 4).

(3) $$\begin{array}{r} FF=\frac{P_{max}}{AJ_{sc}V_{oc}} \end{array}$$

(4) $$\begin{array}{r} \eta =\frac{J_{sc}V_{oc}}{I_{in}}FF \end{array}$$

The performance of the solar cells is directly related to fundamental material properties. For example, the generation of free electrons and holes depend on the HOMO_D_ of the donor and on the LUMO_A_ of the acceptor, as will be explained in detail in section Physical Processes at the Donor/Acceptor Interface. This energy gap between the HOMO_D_-LUMO_A_ (E_g,DA_ in Figure 1B) is also the main limiting factor for the open circuit voltage (*V*_*oc*_) of the device. The short-circuit current density (*J*_*sc*_) depends on the charge generation efficiency in the donor/acceptor blend and the probability that these charges percolate through the blend to be collected by the electrodes. Finally, the third relevant parameter directly related to the solar cell efficiency is the fill factor (*FF*), which is affected by the shunt and the series resistances. It should be emphasized here that the device's resistance does not only depend on the conductivity of the layers (active and the transport layers) but especially on the interface resistances. The fill factor is directly related to the efficiency of the charge extraction from the solar cell. In a microscopic point of view, the higher is the loss of the charge carriers by bimolecular recombination, the lower is *FF*. In BHJ-type solar cells, the bimolecular recombination occurs mainly at the donor-acceptor interfaces in the active layer and also at the interfaces of the intermediate layers.

Another important property of the materials is the bandgap. The absorption spectrum of the polymer solar cells depends upon the bandgap of the donor polymer (E_g_ in Figure 1B), which is roughly the difference between its HOMO and LUMO. Assuming that the electron acceptor is the same and has negligible light absorption (as in the case of C_60_ derivatives), using a donor polymer with a lower band gap increases the solar spectrum absorption and consequently increases the photocurrent and the power conversion efficiency of the solar cell. On the other hand, E_g,DA_, the so-called effective bandgap, will also be reduced with the donor E_g_, as a minimum offset must exist between LUMO_D_ and LUMO_A_ for electron transfer. Thus, the open circuit voltage is also reduced, lowering the efficiency of the solar cells. Therefore, for obtaining a maximum efficiency, there is a trade-off between lowering the polymer bandgap for increasing the absorption of the solar spectrum, but still keeping a reasonable open circuit voltage that is determined by E_g,DA_. With these assumptions, the optimum bandgap for the donor polymer should be around 1.5–1.8 eV for a single junction cell (Scharber and Sariciftci, 2013).

An additional approach to improve the device efficiency is the use of the tandem junction BHJ solar cells, as shown in the Figure 1D. As the two junctions are connected in series, the current through both must be the same, and therefore, the overall power from the tandem cell increases due to the sum of the voltages from each junction (You et al., 2013). A good balance of the generated currents from each individual junction (if operated separately) guarantees that both contribute significantly to the total power generated by the device in the tandem configuration. The major advantage of a tandem cell is that its multiple active layers consist of donor polymers with different bandgaps, leading to light absorption at different portions of the solar spectrum. In the case of a single junction solar cell, photons with an energy much larger than the bandgap (E_g_) result in highly excited carriers (or excitons) that lose their excess energy to lattice phonons or molecular vibrations, thereby cooling to the bandgap edge. Such energy loss is usually referred to as thermalization loss. Therefore, reducing the polymer bandgap increases the light absorption, but the absorbed higher energy photons would lead to larger thermalization losses. Thus, the overall efficiency increase may not be as high as expected by the increased absorption, since the total cell voltage is limited by the low D-A bandgap, E_g,DA_. In contrast, for a tandem cell, the absorption of the incident light with different wavelengths by separate cells increases the overall device efficiency by reducing thermalization losses. The first active layer with a larger bandgap polymer absorbs higher energy photons, which leads to a higher *V*_*oc*_ for that first cell. The second active layer has a low bandgap polymer that absorbs unused photons by the first cell and generates an additional voltage. Since these photons have lower energies, their thermalization losses are also kept small in the active layer of this second, low bandgap polymer cell.

Although there has been a remarkable increase in cell efficiency in about a decade of research and development of new materials, fabrication procedures and architectures, as indicated by Table 1, there is also a huge variation in the efficiencies reported across the literature for the same materials under similar fabrication conditions. This clearly points toward the lack of reproducibility of the efficiencies, which may result from variations in material purity, solvent choice, slight differences in fabrication conditions and use of additives to improve BHJ morphology. These issues will not be discussed in detail here, and we refer the reader to other reviews in the literature (Coakley and McGehee, 2004; Spanggaard and Krebs, 2004; Brabec et al., 2005; Coakley et al., 2005; Janssen et al., 2005; Krebs, 2005; Shaheen et al., 2005; Bundgaard and Krebs, 2007; Mayer et al., 2007; Rand et al., 2007; Kroon et al., 2008).

Another important aspect for the commercial viability of organic solar cells is their stability in the ambient atmosphere. In order to prevent the degradation of the solar cells, the factors responsible for the degradation must be understood in detail. It has been reported that the materials used in the fabrication of polymer solar cells (especially the active layer materials and metallic electrodes) undergo chemical interactions with oxygen and moisture present in the ambient atmosphere (Jørgensen et al., 2008). The mechanism by which the oxygen and moisture react with the donor polymer is different for each material (Matturro et al., 1986; Jørgensen et al., 2008). However, such chemical degradation not only alters the material in the bulk film but can also introduce changes at the interfaces that lead to poor device efficiency. For example, the degradation of the aluminum electrode may be caused by the acceptor fullerene derivative PCBM which has high affinity for the electrons which makes them prone to react with metal electrodes (Lögdlund and Brédas, 1994). This degradation of the aluminum electrodes leads to ineffective charge transfer across the metal/active layer interface, whose effects will be briefly discussed below and in more detail in section Injection and Extraction Barriers.

Ineffective charge transfer across the various interfaces within the device is one of the most important factors responsible for the reduced performance of polymer solar cells. It decreases device parameters like open circuit voltage, short circuit current density and the fill factor, and thus the overall efficiency of the device. This issue is governed by the type and quality of various interfaces between the layers of the solar cell. Appearance of the so-called “S-kink” in the J-V characteristics (see Figure 2B) is well-known, strongly reducing the fill factor (FF) and hence the efficiency of the BHJ solar cell. S-kink has been observed in many organic BHJ devices and has been attributed to interface morphology as well as mismatched injection and extraction barriers across the interface (Kumar et al., 2009; Tress et al., 2011). Apart from poor quality of the interfaces, other factors responsible for the appearance of S-kink include oxygen doping, presence of organic impurities, vertical phase segregation, reduced surface recombination, formation of charge dipole, etc. (Gusain et al., 2016). Therefore, preparation of high-quality BHJ solar cells requires robust characterization to ensure that key physical parameters, such as layer thickness, interfacial quality and morphology are well-controlled.

Indeed, the other type of the degradation that solar cells undergo is the morphological instability of the materials in the various layers of the device. The thin film materials may undergo morphological changes at room temperature during the period of their operation (Wu et al., 2011). Furthermore, these changes in the active layer morphology may also depend on the process and the method of the fabrication of the solar cells (Matturro et al., 1986; Lögdlund and Brédas, 1994; Dam et al., 1999; de Jong et al., 2000; Winder and Sariciftci, 2004; Mühlbacher et al., 2006; Norrman et al., 2006, 2010; Vanlaeke et al., 2006; Hou et al., 2008; Jørgensen et al., 2008; Park et al., 2009; Tsai et al., 2010; Chu et al., 2011; Tress et al., 2011; Wu et al., 2011; Albrecht et al., 2012; He et al., 2012, 2015; Li et al., 2012; Gusain et al., 2013; Lu et al., 2013; You et al., 2013; Zhang et al., 2013; Zhou et al., 2013, 2015; Chi et al., 2014; Liu et al., 2014b; Liu S. et al., 2015). Differences in the active layer morphology induced at interfaces, and their possible effects on device performance will be discussed in section Interfacial Morphology.

We will now present a description of a few physical processes occurring at interfaces that influence the device performance.

## Physical Processes at the Donor/Acceptor Interface

**Figure 3:**
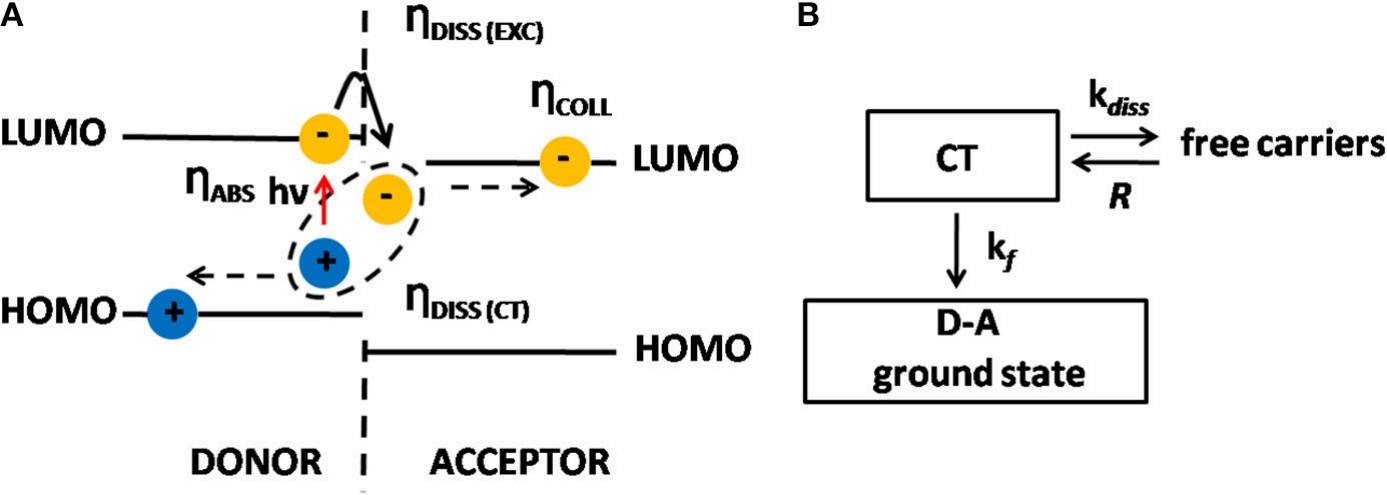
**(A)** Energy diagram showing the dissociation of excitons into charge carriers via CT states. **(B)** Processes involving the CT states and their rates: *k*_*f*_ is the decay rate to the ground state, *k*_*diss*_ is the dissociation rate of the CT state into free charges and *R* is the recombination rate of free charges back to the CT state.

Bulk heterojunction organic solar cells (BHJ-OSCs) differentiate from other thin-film solar cells because they have an active layer comprised of a biphasic nanostructured blend of a donor conjugated polymer and acceptor molecules, as depicted in Figure 1A. The absorption of one photon by the polymer generates one exciton, which diffuses toward the donor-acceptor (D-A) interface. The morphology of this layer is such that the D-A phase separation is in the range of 10–20 nm, which is shorter than the exciton diffusion length. In BHJ devices the interfacial D-A area is then vastly increased when compared to a traditional D-A bilayer device. This is a tremendous advantage because the efficiency in converting each exciton into a pair of free electron and hole is almost 100%. This conversion, however, is not a direct process but instead is intermediated by a Charge Transfer (CT) state formed when the exciton dissociates; i.e., the acceptor captures the electron of the exciton leaving the hole in the polymer, and this pair of charge carriers remains Coulombically bound, which is the CT state (Figure 3A). From then on, the generation of free carriers, their recombination and also collection by the electrodes is triggered, as is described below.

Since positive and negative charges are Coulombically bound, the hole being located in the HOMO of the polymer and the electron in the LUMO of the acceptor, they can either separate in free carriers (dissociation) or recombine (geminate recombination). If they separate, there is a chance of recapture in the CT state. In this sense, CT state intermediates the process of recombination and generation of free charge carriers (Braun, 1984). Based on the scheme outlined in Figure 3B, we can infer that the probability *P* that CT dissociates into free charge carriers is given by Equation (5):

(5) $$\begin{array}{r} P=\frac{k_{diss}}{k_{f}+k_{diss}} \end{array}$$

where these rates are shown in Figure 3B.

**Figure 4:**
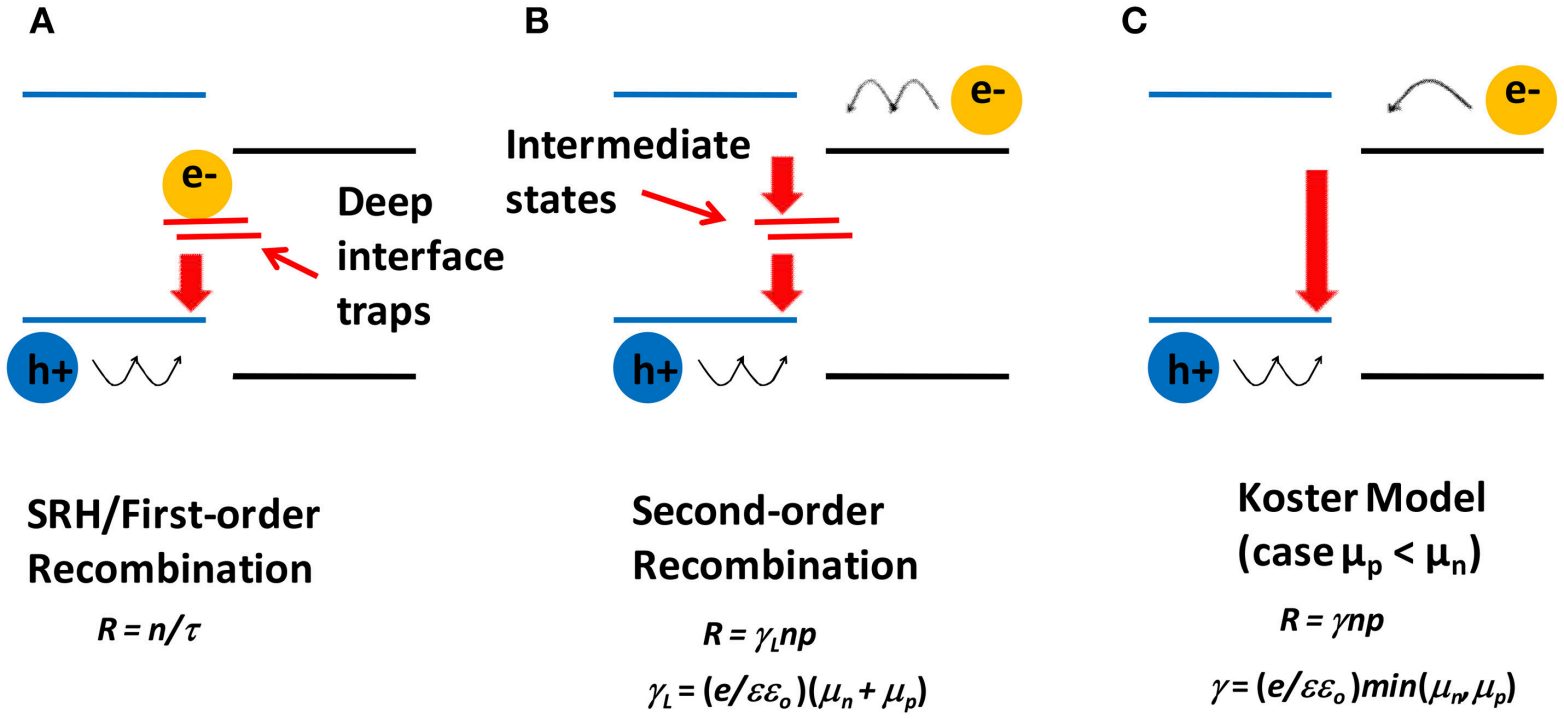
Schematic diagrams of different charge recombination mechanisms. **(A)** First-order recombination by interfacial deep traps. For example, electrons are trapped and holes need to diffuse to the interface (represented by wiggly arrows). **(B)** Second-order interfacial recombination, where both electrons and holes need to diffuse to the interface. **(C)** Koster model of recombination, which is limited by the lower carrier mobility (slower holes for the case represented here).

Once the dissociation of CT occurs, electrons move in the acceptor phase and holes in the donor phase toward the respective electrodes under the action of the internal electric field generated by the built-in voltage. However, in this journey, carrier loss is generally very large due to bimolecular recombination, which can only take place at the D-A interface. Several models have been proposed for molecular recombination in the active layer of BHJ-OSCs (Hall, 1951; Shockley and Read, 1952; Koster et al., 2006; Pivrikas et al., 2007; Deibel et al., 2009; Hilczer and Tachiya, 2010). From the point of view of the recombination kinetics, the most plausible ones are the first order and the second order kinetics. The first-order recombination kinetics for the D-A interface arises when one of the carriers is trapped in a deep localized state near the interface, forming a recombination center for the oppositely charged carrier. Figure 4A shows schematically this type of recombination, which is similar to the Shockley-Read-Hall (SRH) process (Hall, 1951; Shockley and Read, 1952), in which the recombination term *R* is given by *R* = *n/*τ, where *n* is the concentration of the free charge carrier and τ is its lifetime. Second order recombination is explained when a positive and a negative carrier arrive concomitantly to the interface, where they are captured by localized states and then annihilated by recombination, as illustrated in Figure 4B. In this case, the recombination, by the symmetry of the process, is of the Langevin type, that is *R* = γ*np*, where *n* and *p* are the respective concentrations of positive and negative carriers, and γ is the recombination coefficient. The Langevin recombination coefficient γ_*L*_ is defined as γ_*L*_ = (*e/*εε_*o*_)(μ_*n*_ + μ_*p*_), where *e* is an electron charge, ε is a dielectric constant, ε_*o*_ is a vacuum permittivity, μ_*n*_ and μ_*p*_ are the mobilities of electrons and holes, respectively. However, Pivrikas et al. (2007) observed a reduction in the recombination rate by orders of magnitude for BHJ-OSCs based on P3HT:PCBM. This reduction is often taken into account by multiplying Langevin's recombination rate by a prefactor β (γ = βγ_*L*_), where β varies within the range from 10^−3^ to values close to 1. This ratio β is known as reduced Langevin recombination and depends on the materials of the active layer. Koster et al. (2006) showed that indeed Langevin recombination does not fit accurately for photovoltaic responses of BHJ-OSCs, and they put forward another value for the recombination coefficient, without abandoning the essence of Langevin's concepts. They define the new coefficient as γ = (*e/*εε_*o*_)*min*(μ_*n*_,μ_*p*_), i.e., the recombination coefficient is now controlled by the mobility of the slowest carrier. This picture is outlined in Figure 4C. Another interpretation for β was given by Deibel et al. (2009), who assumed that the bimolecular recombination depends on the spatial profile of charge carrier concentrations. Their approach also explained satisfactorily the variation of β with temperature. Hilczer and Tachiya developed a model based on a formalism that took into account that the recombination occurs when the charge carriers are a non-zero distance to each other (Hilczer and Tachiya, 2010). This approach fitted quite well the temperature dependence of β.

We now turn our attention to phenomena occurring at those many flat (extended) interfaces within the OSCs. In the next section we will address how and why the BHJ morphology may change at the interfaces of the active layer with electrodes or interlayers. This may have an impact on both charge generation/recombination near these interfaces and charge transport across them.

## Interfacial Morphology

As discussed in the previous section, the blend morphology at the micro/nanoscopic scale is a critical factor determining the device efficiency for BHJ polymeric solar cells (Quiles et al., 2008; Felicissimo et al., 2009; Germack et al., 2009; Xu et al., 2009; Wang et al., 2012; Mauger et al., 2013; Mor et al., 2014b). With a poor blend morphology, with phase separation in the range of hundreds of nanometers or more, the exciton diffusion to the donor-acceptor interface may not be complete, reducing the charge generation efficiency. Furthermore, a poor blend morphology may lead to reduced percolation pathways to the electrodes within each semiconducting material of the active layer, affecting the charge transport properties and thus increasing the possibility of charge recombination and reducing their collection efficiency by the electrodes. All these factors contribute to lower device efficiency. In addition, several factors related to the blend morphology like interfacial area, packing of the molecules in each phase and variation of the composition among the domains within the blend are also important in governing the device performance.

However, it has been reported that the interfacial morphology can be significantly different from the bulk film morphology due to the variation in the composition of the active layer (and also the electrodes) along the thickness of the device, leading to poor charge transport across the interfaces (Germack et al., 2009; Wang et al., 2012; Mor et al., 2014b). Such variation in the composition profiles has been attributed to different factors, which will be discussed below. It has also been shown that such compositional profile near interfacial layers may have a significant effect on the device efficiency (Germack et al., 2009; Wang et al., 2012; Mor et al., 2014b). A few examples will be described in section Control of Interfacial Morphology and Its Effect on Device Performance. Furthermore, since the interfacial morphology is also directly related to the “bulk” blend morphology, it is thus important to control and improve the blend morphology for a better interfacial morphology, with a corresponding impact both on the BHJ properties (charge transport and generation efficiency) and on the interfacial properties, which in turn affect the charge collection efficiency by the electrodes.

### Governing Factors for Vertical Phase Segregation

The phase segregation of the domains in the BHJ is dependent upon several factors and the fabrication procedures, such as appropriate choice of the solvents, drying rate of the spin coated films and thermal and/or vapor annealing of the blends (Chirvase et al., 2004; Kim et al., 2005, 2006; Li et al., 2005; Mihailetchi et al., 2006; Zhao et al., 2007; Quiles et al., 2008). Furthermore, it has been recognized that various factors may also contribute to the formation of a non-homogeneous blend morphology within active layer film, leading to a vertical profile of the blend composition along the device thickness. This so-called vertical phase segregation has been observed in different combinations of polymers and acceptor molecules and has been reported to arise from the differences in the solubility/surface energies of the component materials within the blend, the dynamics of the spin coating process (Heriot and Jones, 2005; Björström et al., 2007), the diffusion of oxygen and moisture from the ambient atmosphere, interactions between the organic materials and substrates, and the spontaneous creation of surface wetting layers which may lead to changes in the composition of active layers (van Zanten et al., 1996; Arias et al., 2002; Björström et al., 2005; Reyes et al., 2005; Choulis et al., 2006; Goffri et al., 2006; Quiles et al., 2006, 2008; Mor et al., 2014b).

In a simple picture, this vertical phase segregation occurs during the solvent evaporation process due to the differences in the surface energies of the polymer and the fullerene molecules in the blend, and allows for the morphology of the films to reach a thermodynamically more favorable state. For example, in the case of P3HT/PCBM, it has been reported that the surface energy of P3HT is lower than that of PCBM, which allows it to accumulate at the free surface to reduce the overall energy (Björström et al., 2005; Heriot and Jones, 2005). Furthermore, charge transfer interactions at an interface may play a role in causing the vertical composition profile. An XPS analysis has suggested that there is a significant binding energy shift (~0.5 eV) to lower binding energy for Cs after spin-coating an ultra-thin PCBM layer on the top of a Cs_2_CO_3_ interlayer, showing a charge transfer from the interlayer to the PCBM (Xu et al., 2009). This charge transfer results in the formation of a dipolar region between the Cs_2_CO_3_ and PCBM layer, which may favor enrichment of PCBM toward the Cs_2_CO_3_ as compared to P3HT (Xu et al., 2009). Apart from this interaction, the formation of a strong dipole between Cs_2_CO_3_ and the ITO substrate has been shown to occur and may also play a role in inducing vertical phase segregation (Huang et al., 2008). Such dipole—induced-dipole interaction has also been suggested to contribute toward the enrichment of PCBM at the blend/Cs_2_CO_3_ interface (Huang et al., 2008). Thus, the vertical phase segregation within the blend of the device is shown to occur due to the contribution from both the differences between the surface energies of the polymer and the fullerene molecules in the blend, as well as the charge transfer from the substrate to the fullerene molecules.

### Characterization of the Interfacial Morphology

The level of vertical phase segregation of the domains within the blend can be determined by an appropriate characterization of the interfaces using different techniques. Various non-destructive techniques like X-ray photoelectron spectroscopy (XPS), near edge X-ray absorption fine structure spectroscopy (NEXAFS), X-ray reflectometry (XRR), and neutron reflectometry (NR) have been employed for the analysis of the vertical composition profiles within the active layer of the devices (Paci et al., 2005; Germack et al., 2009, 2010; Kiel et al., 2010; Orimo et al., 2010; Parnell et al., 2010; Chen et al., 2011b; Lee et al., 2011; Rochester et al., 2012; Gusain et al., 2016). However, some steps of the sample preparation for these techniques are either destructive, requiring isolation of layers from the substrates, or are carried out on different interfaces from those of actual devices, such as at the air/blend interfaces or anodic covered interfaces, or even require surface modification of the substrates (Chen et al., 2011a; Mor et al., 2014b). For example, since the probing depth of the XPS technique is limited to 6–8 nm due to the short mean free path of the photoelectrons, the profile analysis of the interfaces at depths below this limit not possible (Xu et al., 2009). Therefore, in order to analyze buried interfaces by XPS, the films can be lifted off from different substrates and then transferred to a conductive substrate with the desired surface on the top side for the XPS analysis (Xu et al., 2009).

In order to probe the vertical composition profile, a surface-sensitive technique such as XPS can be combined with the destructive technique of ion sputtering. The XPS spectra initially probe the topmost device/air interface, but these topmost layers are gradually sputtered away, so that the exposed surface being probed by XPS gradually moves toward the bulk of the original device, until reaching the substrate. However, it has been reported that the measured depth profile with such approach may be different from the actual profile, due to unforeseen changes induced by the sputtering process like chemical bond breaking, preferential sputtering, interface mixing or roughening (MacKay et al., 1991; Song et al., 2001; Turak et al., 2002; Kwoka et al., 2006; Xu et al., 2009).

In contrast, a few other studies have been performed in a non-invasive manner. These techniques have revealed the oxidation of the aluminum electrode at its interface with the organic material by the oxygen diffused through the electrode. This resulted in the formation of an insulating layer of aluminum oxide, which thereby acted as a blocking layer, degrading the device performance (Norrman and Krebs, 2006; Wang J. C. et al., 2011; Wagner et al., 2012) and yielding S-kinks in the J-V curves for the solar cells (Wagenpfahl et al., 2010; Ecker et al., 2012; Mor et al., 2014a). Other techniques like energy-filtered transmission electron microscopy (EF-TEM) and XPS have also been employed to examine the cathode interface in P3HT/PCBM solar cells (Mor et al., 2014b). In contrast to the previous reports, they show that such formation of aluminum oxide occurs preferentially at the cathode interface irrespective of the device fabrication conditions, in ambient or inert environmental conditions. However, it is argued that such a thin aluminum oxide layer acts as a dielectric sheet between the electrode and the active layer and improves the charge transport across the interface by preventing the formation of electronic barriers for charge extraction (Mor et al., 2014b).

Finally, non-linear optical spectroscopic techniques such as second-harmonic generation (SHG) and sum-frequency generation vibrational spectroscopy (SFG) have a good potential to investigate interfaces in organic electronics (Motti et al., 2014; Maia and Miranda, 2015), but have not been extensively used so far. Since they rely on broken inversion symmetry, they could, in principle, probe the vertical phase segregation in a photovoltaic blend, since in a homogeneous blend there is inversion symmetry (on the scale of optical wavelengths), while at an interface this symmetry is broken. Therefore, the detected signals are generated only at interfaces, and the SFG vibrational spectroscopy could in principle identify and quantify the surface composition, thus shedding light on interfacial segregation of a component. This issue of interfacial segregation of additives has already been extensively investigated by SFG spectroscopy for non-conjugated polymers (Lu et al., 2017; Zhang, 2017), but not really explored for conjugated polymers and blends.

### Control of Interfacial Morphology and its Effect on Device Performance

The effect of the blend morphology on the device performance can be related to its influence on the charge generation and transport properties across the blend, and to its behavior at interfaces with electrodes or HTLs/ETLs. It has been reported that the charge transport in the blend depends directly on the effective vertical as well as the lateral phase segregation of the polymer and fullerene domains within the blend (Quiles et al., 2008). The charge extraction process is improved when the donor polymer domains segregate toward the anode interface and the acceptor fullerene domains segregate toward the cathode interface (Heutz et al., 2004; Snaith et al., 2004; Xue et al., 2005; Quiles et al., 2008). Such combination of vertical and lateral phase segregated morphology favors the charge transport across the blend and across the interfaces, thus improving the device performance.

As an example, a non-destructive study employed XPS and atomic force microscopy (AFM) to study the interfaces between the blend and electrodes for P3HT/PCBM solar cells (Xu et al., 2009). It was revealed that due to the vertical phase segregation, the domains of P3HT are enriched at the free surfaces, while the fullerene derivatives accumulate at the organic/substrate interfaces. Such vertical phase segregation is advantageous for the inverted device structure, consisting of ITO/Cs_2_CO_3_(non-annealed)/P3HT:PCBM/V_2_O_5_/Al, since it yields the accumulation of fullerene derivatives at the organic/substrate (cathode) interface for efficient electron extraction, and P3HT enrichment at the organic/anode interface, allowing good hole extraction at that electrode (Xu et al., 2009). Apart from improved charge extraction, the inverted device structure allows for the replacement of both the low work function metal cathode and the PEDOT:PSS HTL, which are responsible for the degradation of the device, and thus improving the device lifetime (Sahin et al., 2005; Watanabe and Kasuya, 2005).

As discussed above, the vertical phase segregation may lead to efficient charge extraction at electrode/blend interfaces. However, such effect is asymmetric in nature, as the P3HT and PCBM have different transport properties through the blend. For example, it has been shown that P3HT does not block the electron conduction but PCBM is known to significantly block the hole conduction through the blend (Germack et al., 2009; Wang et al., 2012; Mor et al., 2014b). Thus, the reverse process, with PCBM enrichment at the anode/blend interface and P3HT enrichment at the cathode/blend interface, would be expected to also degrade the device performance due to the hole blocking property of PCBM at the anode. Nevertheless, this was not observed (Wang H. et al., 2011). The study employed the application of delamination and transfer method to flip the P3HT/PCBM active layer of the device, thus reversing the interfacial segregation of the active layers to investigate their contribution to the device performance. With such reverse interfacial segregation of the active layer, it was expected that the device would exhibit high series resistance due to the hole blocking by PCBM at the anode, leading to a reduced device performance. However, it was found that the dark and illuminated characteristics of both the reverse and as deposited devices were comparable, suggesting that interfacial segregation of the active layers may not significantly influence the device performance, as long as the active layer/anode interface does not comprise a continuous layer of the fullerene acceptor that hinders charge extraction.

**Figure 5:**
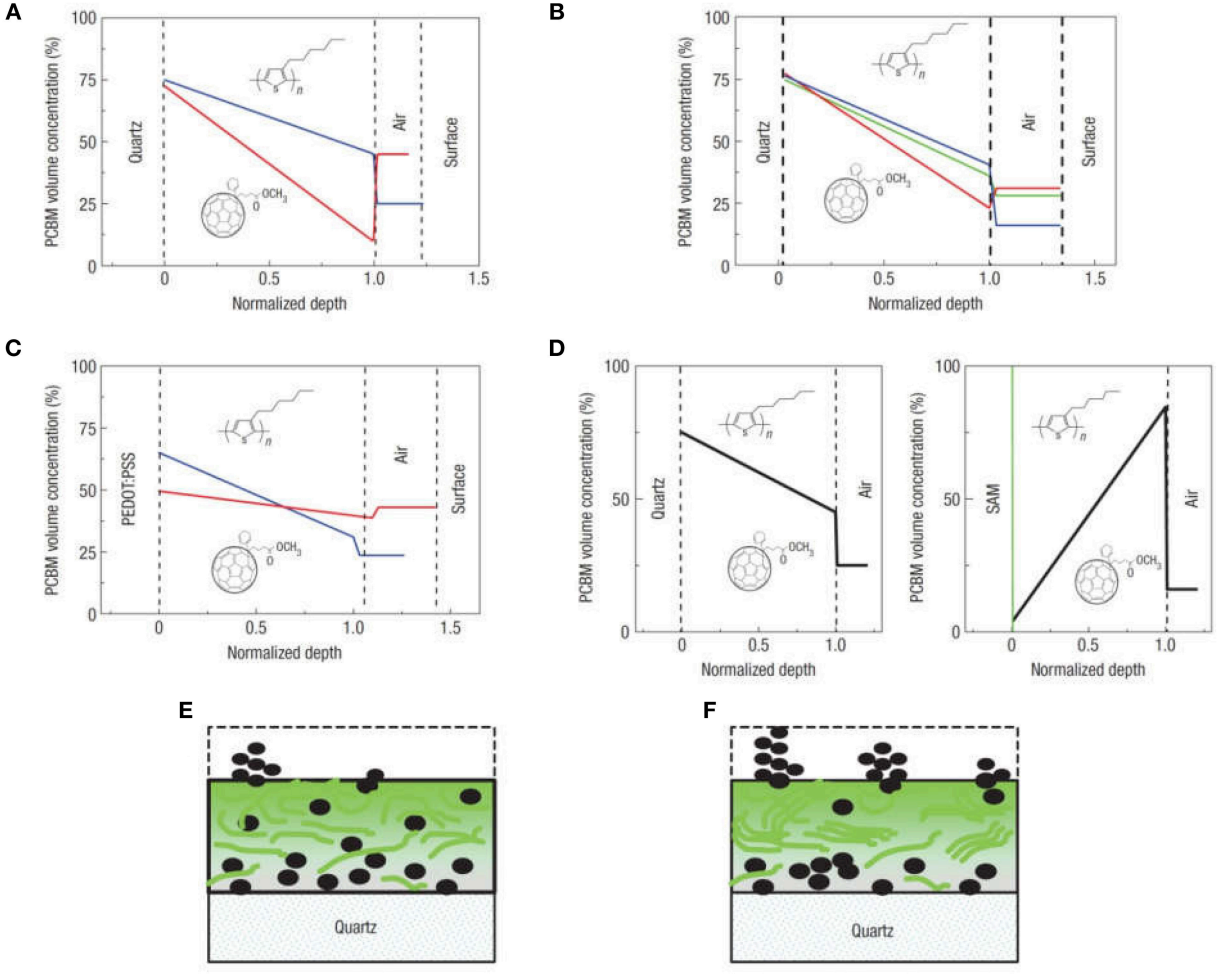
Vertical composition profiles in P3HT:PCBM films. **(A–D)** PCBM concentration profiles obtained from analysis of P3HT:PCBM blend films: **(A)** spin coated on fused silica before (blue) and after (red) thermal annealing **(B)** spin coated on fused silica for 60 s at 5,000 r.p.m. (blue), 3,000 r.p.m. (green) and 700 r.p.m. (red) (the blend films here were typically ~50 nm thick) **(C)** spin coated on PEDOT:PSS-coated fused silica before (blue) and after (red) vapor annealing **(D)** spin coated on fused silica (left) and on a Si wafer (with native oxide) precoated with a hydrophobic self-assembled hexamethyldisilazane monolayer. Typical PCBM distributions before **(E)** and after **(F)** vapor or thermal annealing. Reprinted by permission from Macmillan Publishers Ltd (Quiles et al., 2008), copyright (2008).

The degree of the vertical phase segregation depends on the nature of the substrate and spin coating conditions, and can also be controlled using thermal annealing or by changes in the blend-substrate interface by the modification of the substrate surface energy (Quiles et al., 2008). This is shown in Figure 5, where the depth profile of PCBM in the blend was measured by ellipsometry, and investigated under several fabrication conditions. Interestingly, the segregation of PCBM toward the substrate is smaller for the blend spin-coated on PEDOT:PSS, and is reduced by thermal annealing (Figure 5C). In contrast, it is larger for the blend spin-coated on fused silica substrates and is enhanced by thermal annealing (Figure 5A). In this latter case, the depth profiles suggest that the free surface is enriched of PCBM clusters, a fact that is confirmed by *in situ* optical microscopy during thermal annealing. Furthermore, as can be seen in Figure 5D, the deposition of a molecular self-assembled monolayer of hexamethyldisilazane on the substrate modifies its surface energy, leading to a change in the direction of compositional gradient of PCBM in the blend, with the film enriched of P3HT domains toward the substrate while PCBM is displaced toward the free surface (Quiles et al., 2008).

One study has used NR for analyzing the compositional profiles of the layers along the depth of the device to reveal the influence of the different capping metal electrodes as well as the addition of the solvent additive nitrobenzene on the thermal stability of the device (Mauger et al., 2013). The same study has also further reported that changes in the compositional profiles due to the thermal annealing can be due to a specific charge transfer or doping process occurring between the metal electrodes and PCBM due to differences in their Fermi Levels (Mauger et al., 2013). The NEXAFS analysis revealed that the PCBM undergoes chemical changes after the thermal annealing depending upon the metal electrode. It has been shown that doping and charge transfer occurs more strongly with Ca than Al, leading to differences in the vertical phase segregation and the compositional profiles depending upon the type of the electrode (Mauger et al., 2013). Apart from the thermal annealing of the device, the addition of the small concentrations of solvent additives is usually employed for controlling the typical size of phase separated domains in BHJ devices, and thus leading to improved device efficiency (Moulé and Meerholz, 2009; Mauger et al., 2013). It has been reported that, with nitrobenzene as an additive, the device performance of the thermally annealed and non-annealed devices remains similar (Moulé and Meerholz, 2008; Mauger et al., 2013). In contrast, it was found that the solvent additive can lead to a significant reduction in both the vertical phase segregation and the concentration of PCBM at the interface.

Recent studies on the vertical phase segregation in devices using different donor polymers like PBDTTPD, PCDTBT, and PTB7 with the acceptor PC_71_BM have examined the role of the interfacial layers on improvement in the device performance due to the vertical phase segregation (Cao et al., 2016). In devices, using PEDOT:PSS as an HTL, improvement in the device efficiencies have been reported earlier due to vertical phase segregation of the blend (Meng et al., 2014). The study employed an additional interfacial modifier between PEDOT:PSS and the active layer, based upon a low-band-gap polymer with an appended carboxylic acid–based side chain, called PBDTTPD-COOH (Cao et al., 2016). The interfacial layers may influence both the energy level alignment and the vertical phase segregation within the device, which affect the device performance. The study has demonstrated by employing ultraviolet photoelectron spectroscopy (UPS) that the observed difference in the device performance for each of these three donor polymers is not due to a hole extraction barrier for the PTB7 device (Oehzelt et al., 2015). The surface energies of the polymer indicated that at the PBDTTPD-COOH/donor interface there is an enrichment of the donor polymer with respect to the case of the PEDOT:PSS/donor interface, with higher interfacial composition of the PBDTTPD, PCDTBT and PTB7 as 46, 20, and 47%, respectively, as compared to 28, 13, and 41%, respectively, at the PEDOT:PSS interlayer. The study thus suggested that improvement in the performance of the devices, using PBDTTPD and PCDTBT as donor polymers, with the addition of PBDTTPD-COOH interlayer is mainly due to the changes in the blend composition at the PBDTTPD-COOH/donor interface. However, for the PTB7/PCBM devices using the PBDTTPD-COOH interlayer, the interfacial composition remains the same as that of the bulk, which does not lead to significant improvement in the performance of these devices with the added interfacial layer.

However, in another study ZnO/PFN (poly[(9,9-bis(30-(N,N-dimethylamino)propyl)-2,7-fluorene)-alt-2,7-(9,9-dioctylfluorene)]) hybrid films have been shown to be good ETLs, and the improved electron transport properties in this case have been attributed to a formation of PCBM-rich phase within the active layer in close contact with the ZnO–PFN layer (Lee et al., 2016). The formation of such PCBM-rich phase occurs due to the hydrophobic nature of ZnO/PFN layer and its similar surface energy to that of PCBM (~ 22.5 mJ m^−2^), yielding a close electronic contact between the ZnO/PFN layer and the active layer (Lee et al., 2016). Such inverted polymer solar cell devices have shown an improvement in the efficiency from 7.2% up to 9.2% (Lee et al., 2016).

## Injection and Extraction Barriers

### Physical Mechanism and Definitions

The concept of barriers for injection and extraction of charge carriers, or simply, the energy barriers, arises from the difference in chemical potential (or Fermi energy—E_F_) between two dissimilar materials when they are joined together. Upon contact, electron transfer will occur at the interface until the chemical potential becomes the same throughout the materials.

**Figure 6:**
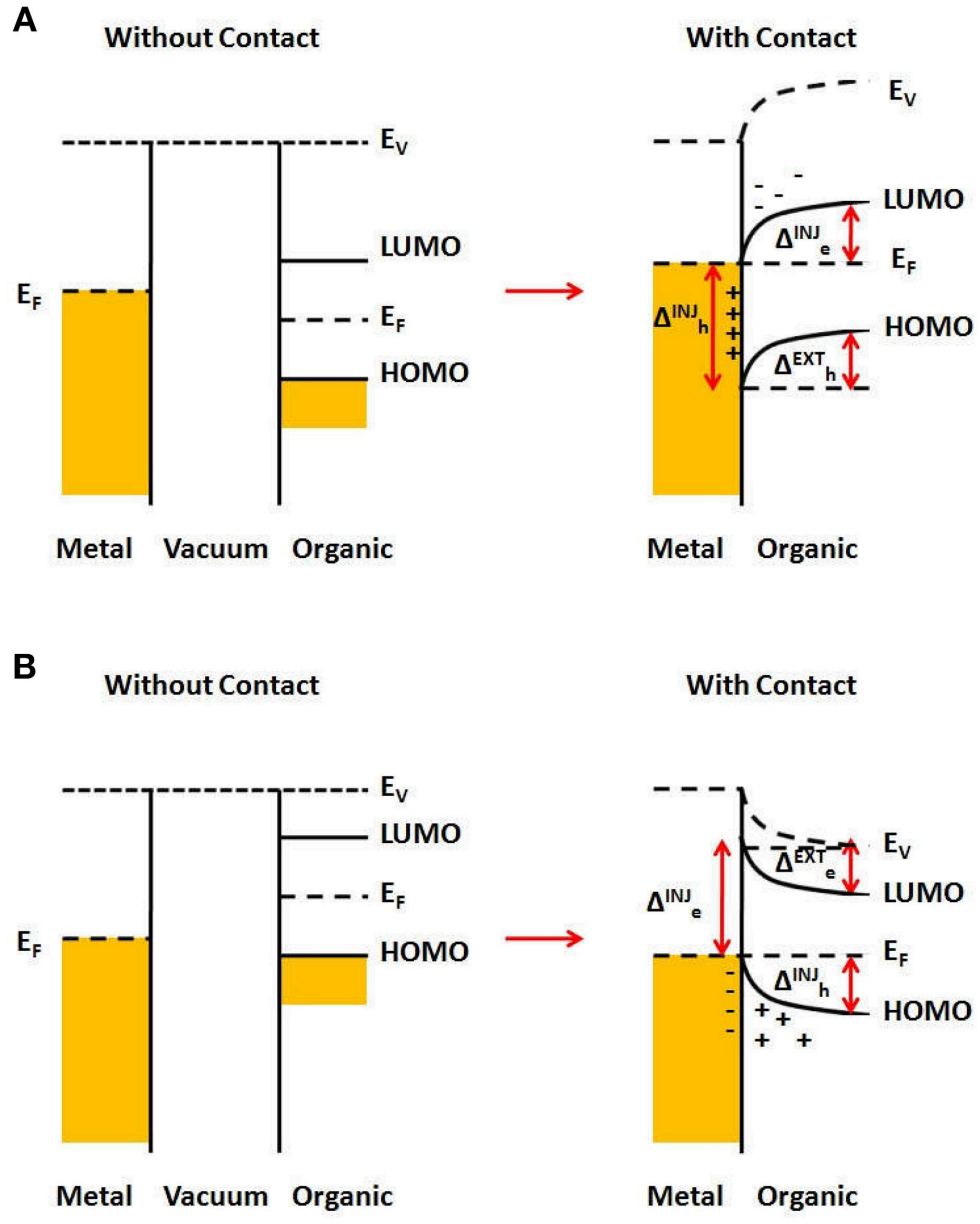
Energy barriers for a metal/organic semiconductor interface. **(A)** Fermi level of metal is below that for the organic semiconductor, before contact. After contact, charge transfer occurs, leading to “band bending” in the organic semiconductor and the formation of energy barriers, labeled as $\Delta_{e\left(h\right)}^{inj\left(ext\right)}$ for injection (or extraction) of electrons (or holes). **(B)** Same as **(A)**, but for the Fermi level of metal below that for the organic semiconductor.

Figure 6 illustrates the typical case for a metal/organic semiconductor interface, where a charge transfer leads to an atomically thin charge density on the metal side, and a distributed charge density in the semiconductor near the interface, known as a carrier accumulation region (or depletion, if the semiconductor is initially doped), whose width depends on the bulk carrier density of the semiconductor, its dielectric constant, temperature and the applied external electric field within the semiconductor material (Sze and Kwok Ng, 2007). In Figure 6A is shown the case of a metal whose Fermi level (before contact) is below that of the organic semiconductor. After joining them, electron transfer from the organic material to the metal will occur, until the Fermi level is the same throughout the materials. This resulting charge distribution will generate a potential that induces a vacuum level shift, together with HOMO and LUMO as well, what is usually known as “band bending.” It can be seen that injecting an electron from the metal to the semiconductor will present an energy barrier $\Delta_{e}^{inj}$, which is larger than that for extracting an electron from the semiconductor to the metal, $\Delta_{e}^{ext}$. On the other hand, there will be no barrier for hole extraction from the semiconductor, while the barrier for hole injection from the metal to the semiconductor is $\Delta_{h}^{inj}$. Similarly, if the Fermi level of the metal (before contact) is higher than that of the semiconductor, there will be electron transfer to the semiconductor LUMO, as illustrated in Figure 6B. In this case, there will be a larger barrier for hole injection from the metal to the semiconductor, $\Delta_{h}^{inj}$, and a smaller barrier for hole extraction from the semiconductor, $\Delta_{h}^{ext}$. On the other hand, there will be no barrier for electron extraction from the semiconductor, while the barrier for electron injection from the metal to the semiconductor is $\Delta_{e}^{inj}$.

In summary, the differences in the HOMO, LUMO, and Fermi levels of the materials used for devices give rise to the energy barriers across the various interfaces. This difference in the energy levels has been termed as an energy barrier if a given carrier must have its energy increased from one material to another, as it crosses the interface. Thus, an energy barrier would depend on the energy levels of the materials, on which carrier is being considered and also on which direction it is being transported. This energy difference may either allow or prevent the movement of charge carriers across the interface depending upon the magnitude of the energy difference and the direction of the movement of the charge carriers. Therefore, in some cases there could be a barrier for carrier injection, but not for extraction across the same interface.

### Implication to the Device Performance

Several studies have investigated in different ways the influence of the injection and extraction barriers on the device performance (Pfeiffer et al., 2003; Maennig et al., 2004; Kemerink et al., 2006; Scharber et al., 2006; Hau et al., 2008; Olthof et al., 2009; Tress et al., 2011; Petersen et al., 2012; Tress and Inganäs, 2013). The effect of the energy barriers in general is to hinder the transfer of the charge carriers across the interfaces, significantly affecting the electrical characteristics of the devices. The physical mechanisms which are related to this charge transfer process and its effect on the J-V curves have been explained in these studies.

**Figure 7:**
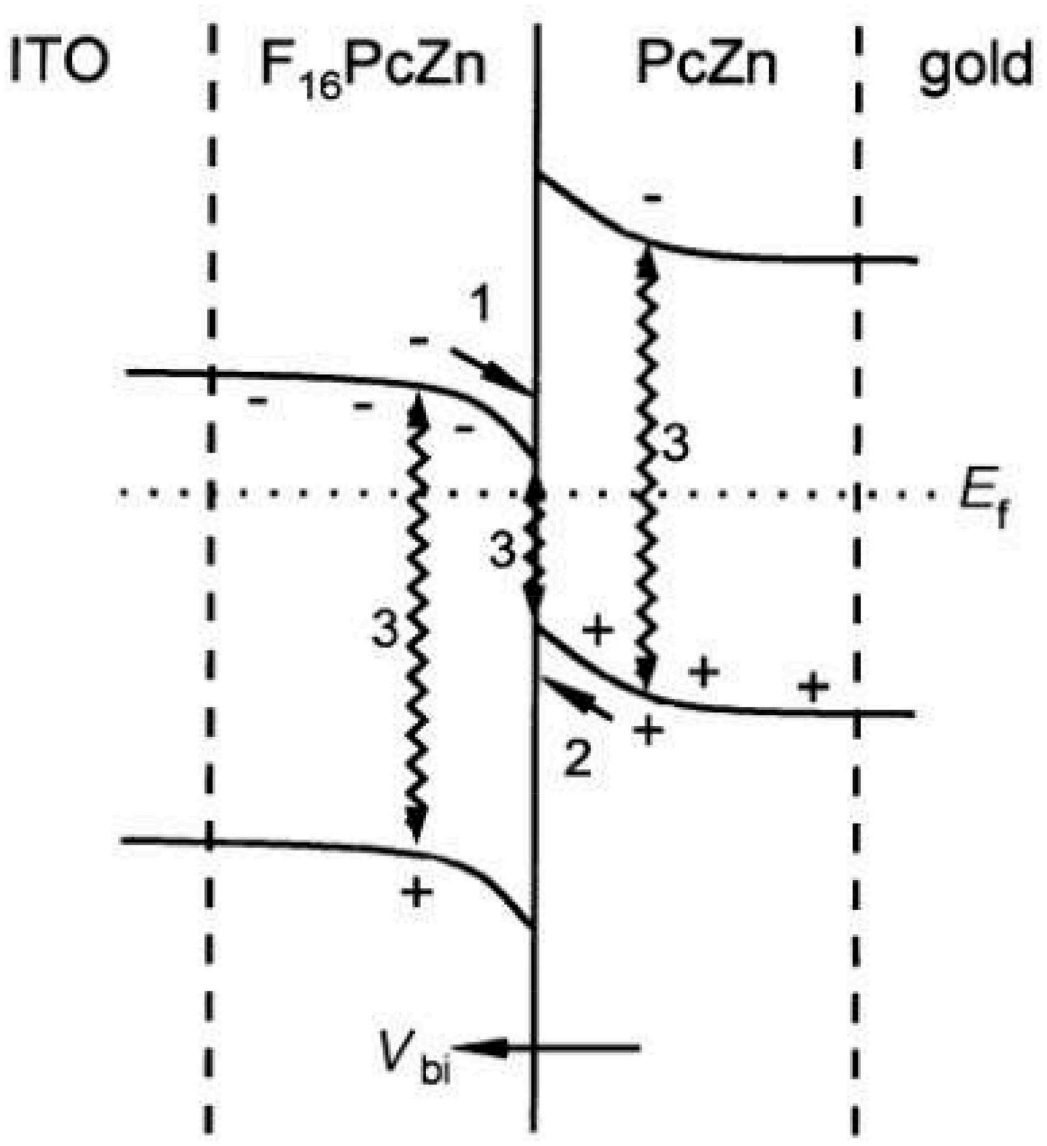
Energy diagram for the contact F_16_PcZn/PcZn after interfacial charge transfer in vacuum with the preferred charge transport direction after exciton dissociation for electrons (1) and holes (2), and the charge recombination pathways (3). Reproduced from Hiller et al. (1998) with permission of The Royal Society of Chemistry.

In a study with bilayer or flat heterojunction devices (FHJs), with PcZn and F_16_PcZn as the donor/acceptor, along with combinations of Au, In and ITO as the electrodes (Hiller et al., 1998), it was found that for certain combinations, the differences in Fermi levels of the different materials led to charge transfer at the junction. These FHJ solar cells consist of the separate layers of donor polymer and acceptor molecule, in contrast with BHJ solar cells where a layer of the blend of the donor polymer and acceptor molecule is deposited to form the nanostructured active layer of the device. The n-type materials form ohmic contacts with the metals having lower work functions, while they yield rectifying contacts with metals having higher work functions. In case of the p-type materials, the opposite effect is exhibited. The study investigated by UPS the formation of a p-n junction with interfacial charge transfer for ITO/F_16_PcZn/ZnPc/Au devices, as can be seen in Figure 7. Photovoltaic response was observed, albeit with a much lower *V*_*oc*_ than expected from the materials Fermi levels. The authors explained this by the several pathways for charge recombination in the junction (wiggly arrows labeled as 3 in Figure 7), which suppressed free carrier concentrations that were not enough to cancel the built-in field *V*_*bi*_ in the device.

**Figure 8:**
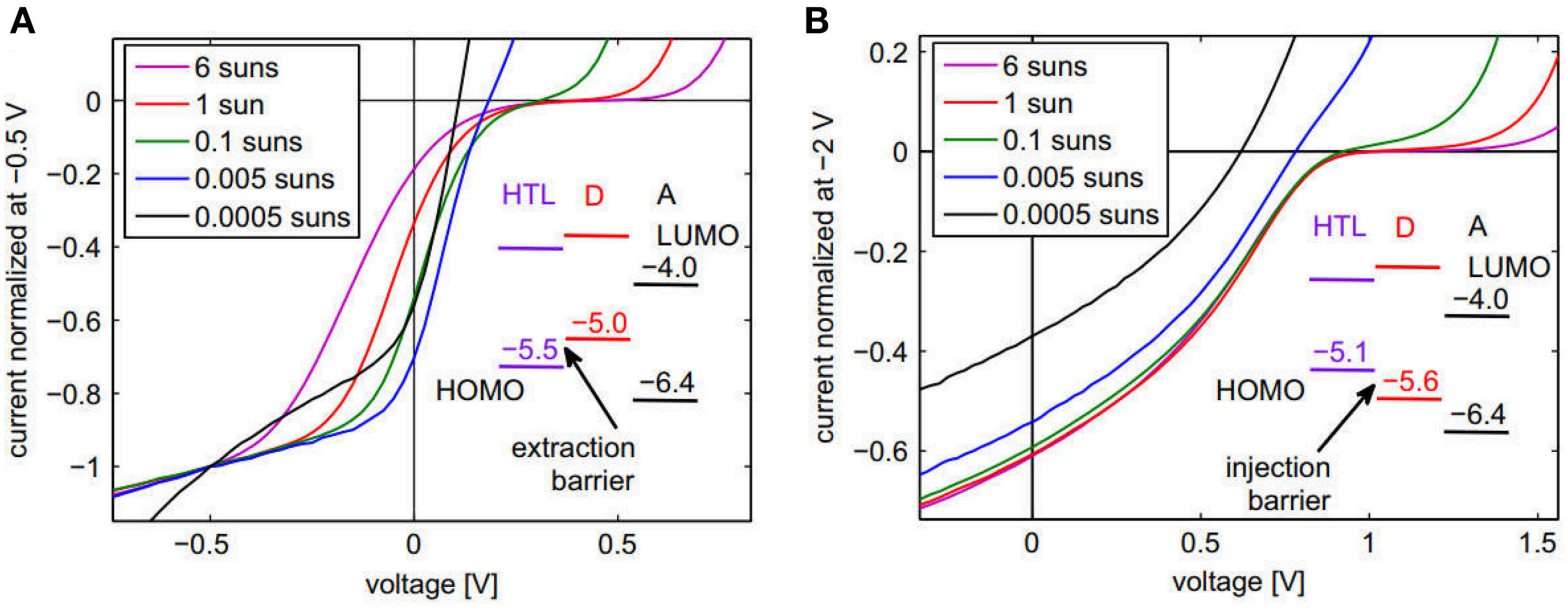
Normalized J–V curves for a series of illumination intensities. **(A)** Device with extraction barrier for holes: ITO/p-doped α-NPB/α-NPB/ZnPc/C_60_/BPhen/Al. **(B)** Device with injection barrier for holes: ITO/p-doped MeO-TPD/MeO-TPD/BPAPF/C_60_/BPhen/Al. Reproduced with permission from Elsevier (Tress and Inganäs, 2013).

As the barriers affect the device performance, it is important to identify the types of barriers present within the device so that a suitable selection of the materials can be done for improving the device performance. One report has shown the application of a simple approach for identifying the barrier in the device (Tress and Inganäs, 2013). The study employed intentional introduction of the barriers with known type and magnitude for the different device configurations shown in Figures 8A,B, corresponding to extraction and injection barriers. In case of extraction barriers for holes, the current-voltage (J–V) characteristics were measured under different light intensities ranging from 0.0005 suns up to 6 suns for the same device. A normalized photocurrent data was plotted, allowing a comparison of the strength of the S-kink, which is more pronounced for the highest intensities. The differences in forward direction (current > 0) have been reported to be mainly an artifact from the normalization. As can be seen in Figure 8A, there are points of intersection (near *V*_*oc*_) of the normalized J-V curves, as *V*_*oc*_ increases with light intensity. They are characteristic of the presence of an extraction barrier, as confirmed by numerical simulations of the J-V curves. The reason for the generation of the S-kinks in the J-V curves is due to accumulation of charges at the donor/HTL interface. This study has shown that at higher intensities, more photogenerated charges pile up at this interface and therefore the strength of the electric field within the HTL increases, while it is reduced at the donor/acceptor interface (Tress and Inganäs, 2013). This leads to higher recombination at the higher light intensities and thus more pronounced S-kinks.

Similarly, in case of injection barriers, the J-V characteristics were measured again under different light intensities ranging from 0.0005 suns up to 6 suns for the same device. In this case no point of intersection was observed, in contrast with the results obtained for extraction barriers (Tress and Inganäs, 2013). These results are characteristics of the presence of an injection barrier in the device. It has been explained that for an injection barrier, there is a low built-in potential (*V*_*bi*_) as compared to the E_DA_ gap. As shown in Figure 8B, for the devices with planar heterojunction architecture, this induces a possibility of higher *V*_*oc*_ as compared to *V*_*bi*_, with the current close to *V*_*oc*_ being completely diffusion-driven against the field. For higher light intensities, this competition between the diffusion and the drift is seen in the form of S-kinks. However, for intensities lower than a threshold value in which *V*_*oc*_ becomes smaller than *V*_*bi*_, the S-kink disappears (Tress and Inganäs, 2013). The study also showed that this method for identifying extraction and injection barriers is also valid for bulk heterojunction solar cells based on P3HT:PC_70_BM configuration (Tress and Inganäs, 2013).

Some earlier studies have reported that the open circuit voltage may be dependent or independent of the choice of the electrodes (Brabec et al., 2001a; Mihailetchi et al., 2003; Cheyns et al., 2008). It has been discussed that the presence of surface charges at the fullerene/metal interface leads to band bending (Brabec et al., 2001a; Cheyns et al., 2008), and the work function of the metal cathode is pinned to the work function of the semiconductor (typically via surface states), independent of whether the work function of the metal is higher or lower than the Fermi level of the semi-conductor. This mechanism is called Fermi level pinning (Brabec et al., 2001a; Cheyns et al., 2008), and would explain why *V*_*oc*_ is independent of the electrode work function. However, it was further shown that such Fermi level pinning is valid only in the cases of ohmic contacts, so that for non-ohmic contacts *V*_*oc*_ depends upon the metal electrode work functions (Mihailetchi et al., 2003).

**Figure 9:**
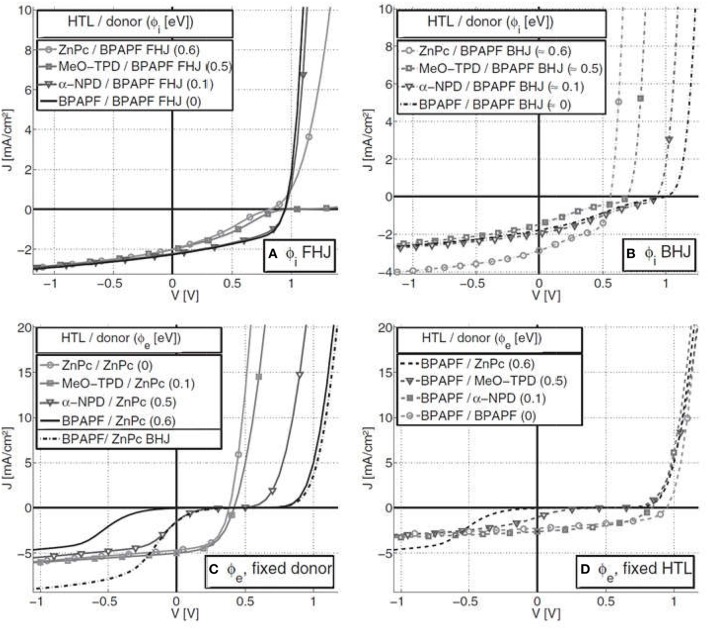
Experimental J-V curves: Injection barriers (ϕ_i_) for the fixed donor BPAPF and varied HTL for **(A)** FHJ and **(B)** BHJ solar cells. Extraction barriers (ϕ_e_) for FHJs with **(C)** varying HTL and fixed donor ZnPc and **(D)** varying donor and fixed HTL BPAPF. In **(C)**, the data for one BHJ (dash-dotted) is also shown. Copyright Wiley-VCH Verlag GmbH and Co. KGaA. Reproduced with permission from (Tress et al., 2011).

Another more recent investigation has reported the effects of injection and extraction barriers on the effective charge carrier mobilities, generation of S-kink and the influence of these barriers on the performance of the both FHJ and BHJ solar cells (Tress et al., 2011). It was found that the energy levels of donor and acceptor in the blend influences formation of the injection/extraction barriers at the HTL/donor interface, and thus the open circuit voltage, the fill factor and the device efficiency (Tress et al., 2011). It was shown that in case of the FHJs, the open circuit voltage (*V*_*oc*_) is linearly dependent upon the gap between the HOMO of the donor and the LUMO of the acceptor (E_g,DA_), but independent of the HTL. Such linear dependence of the *V*_*oc*_ on this E_g,DA_ has already been reported earlier (Scharber et al., 2006). However, for the BHJs, *V*_*oc*_ was also dependent upon the HOMO of the HTL, as displayed in Figure 9B. This result conforms with a prior study where it has been reported that a thin layer of doped organic material acting as the HTL between ITO and the active layer improves the hole injection/extraction, leading to enhanced solar cell performance (Maennig et al., 2004).

Furthermore, for FHJs there is the generation of an S-kink irrespective of the presence of the injection and the extraction barriers (Figures 9A–D). The reason for the generation of the S-kink in the solar cell J-V curve is related to the direction of the electric field in the region of S-kink, which in turn depends upon the type of barrier present in the device. It has been found that in case of the FHJs, in the region closer to *V*_*oc*_ the electric field is reversed and the photocurrent is mainly driven by diffusion. Indeed, earlier studies have also reported such importance of the diffusion gradients for FHJs (Gregg and Hanna, 2003). However, in case of the BHJs, the diffusion gradient for the movement of charge carriers toward the respective electrodes is not present (Tress et al., 2011). As a result, the built in potential is higher in case of the BHJs without barriers, as compared to the device with the barriers and same donor HOMO levels. In any case, for efficient solar cells, good injection/extraction of the charge carriers at the interfaces between the various layers within the device is required.

## Addressing the Challenge of Energy Barriers

After the identification of the actual energy barriers present in the device, it is thus important to address the problem of the misaligned energy levels across the interfaces to facilitate charge transfer and improve the device efficiency. As described in the previous section, the formation of intentional or unintentional blocking interlayers between electrodes and active layers result in a decrease of fill factor, and in extreme cases in S-shaped current–voltage characteristics (Tress and Inganäs, 2013). Several studies have reported different approaches to improve the performance of the devices through proper alignment of the energy barriers (Hau et al., 2008, 2010; Cheng et al., 2010; Hsieh et al., 2010; Chang et al., 2011; Small et al., 2012; Yang et al., 2012, 2014; Chang and Leu, 2013; Kyaw et al., 2013; Jo et al., 2016; Lee et al., 2016; Liu et al., 2017). The two major strategies used so far are described in the sections below: (i) addition of an interlayer that converts a large barrier into two smaller barriers, thus reducing their net effect on the charge transport throughout the device structure; (ii) use of (molecularly thin) dipole layers at an interface to shift the energy levels and reduce the effective barrier height.

### Addition of Interlayers

Earlier attempts to address the issue of the misalignment of the energy barriers have included methods to rationally design the materials of the active layers like donor polymers and the acceptor fullerene in order to facilitate an efficient charge transfer across the interfaces and thus an improved device performance (Small et al., 2012; Kyaw et al., 2013; Liu et al., 2017). However, the introduction of an interlayer between the materials that present an energy barrier, with an intermediate energy level, converts a large barrier into two smaller ones, thus reducing their net effect on the charge transport throughout the device structure. Indeed, a proper choice of the donor polymer and HTL can reduce not only the mismatch between the electrode Fermi level and the HOMO levels of the HTL and donor, but also between the HOMO levels of the HTL and donor (Olthof et al., 2009). A few examples of using such strategy are described below.

The first ones involve the introduction of a cross-linkable n-type acceptor fullerene, incorporation of vertically aligned, cross-linked fullerene nanorods and addition of a combination of Indene-C_60_ Bis-Adduct (ICBA) and cross-linked fullerene interlayer (Cheng et al., 2010; Hsieh et al., 2010; Chang et al., 2011; Jo et al., 2016). The cross linking of the fullerene acceptor functionalized with a dendron containing two styryl groups as thermal cross-linkers (PCBSD, [6,6]-phenyl-C61-butyric styryl dendron ester) yields a robust fullerene interlayer to be used between the metal-oxide buffer layers and the active layer for efficient charge extraction in an inverted solar cell (Hsieh et al., 2010). This prevents interfacial erosion of the fullerene layer against the organic solvent when depositing the active layer. With the addition of such interlayer, initial improvements in the efficiency from 3.5% up to 4.4% have been reported (Hsieh et al., 2010). Furthermore, replacing PCBM as the acceptor fullerene by ICBA in the above studied configuration has shown an enhanced efficiency from 4.8%, with only ICBA, to 6.2%, with both ICBA and interlayer (Cheng et al., 2010). This improvement in the efficiency has been attributed to the higher LUMO of −3.74 eV of the ICBA as compared to −3.90 eV of PCBM. With a higher HOMO-LUMO offset between donor polymer and the acceptor fullerene, a higher *V*_*oc*_ is attainable, leading to higher power conversion efficiency (Cheng et al., 2010). Further studies have shown application of an interlayer of vertically aligned and interpenetrating network of cross-linked fullerene nanorods of PCBSD below the active layer of the inverted device (Chang et al., 2011). Such vertically aligned network of nanorods interlayer provides a larger interfacial area and better aligned energy levels across the interfaces, leading to better charge transport so that improvement in the efficiency from 6.2% up to 7.3% has been achieved (Chang et al., 2011).

Other studies have also reported the application of inorganic buffer layers of TiO_2_ or ZnO between the active layers and the electrodes, which act as electron-transporting/hole blocking buffer layers (Hau et al., 2008, 2010). For example, TiO_2_ has been shown to be a good hole blocking layer and can be used as an electron selective contact in inverted solar cells. With its conduction and valence bands at 4.4 and 7.6 eV respectively, TiO_2_ can be used as a good electron collecting layer between ITO and the bulk-heterojunction blend for inverted solar cell devices (Hau et al., 2008). Application of self-assembled monolayers (SAMs—to be discussed in more detail in section Interface Dipoles) between the TiO_2_ layer and the active layer showed improvement in the device efficiency from 2.80 to 3.78% (Hau et al., 2008). This has been attributed to the better compatibility and removal of the surface trap states on the TiO_2_ surface by SAMs, leading to the reduction of the series resistance and thus an improved performance (Hau et al., 2008). In addition to TiO_2_, several studies have frequently reported ZnO as an excellent material for buffer layer between organic-inorganic interfaces for improving their charge transport (Small et al., 2012; Chang and Leu, 2013; Kyaw et al., 2013; Trost et al., 2013; Woo et al., 2014; Liu et al., 2017), due to its high electron mobility and ease of fabrication of nanostructures of various shapes and sizes. One study has reported improvement in the efficiency from 3.47% up to 4.4% using a combination of C_60_-SAMs and ZnO between the active layer and ITO (Hau et al., 2010). Furthermore, ZnO may also play the role of an optical spacer between the active layer and an Al electrode, placing the active layer away from the region of destructive interference of the incoming and reflected light near the metal electrode and improving the light-harvesting. It will thereby increase the charge generation, improve collection efficiency, and serve as blocking layer for holes and reduce the recombination rate (Hau et al., 2010). More recently, Al doping of ZnO has been reported to boost the device performance by effectively filling the surface defects states of ZnO and reducing its work function because of the electron transfer from Al to ZnO by Fermi level equilibrium (Trost et al., 2013; Liu et al., 2017). The study has reported the deposition of an ultrathin Al layer, thermally evaporated onto annealing-free ZnO electron transport layer, forming a semiconductor-metal nanojunction and facilitating electron extraction and transport (Liu et al., 2017). This contributes to the enhanced short-circuit current density and fill factor, and thus improves the device performance with a reported improvement in efficiency from 7.89% up to 9.81% (Liu et al., 2017).

Another example of interlayer materials are conjugated polyelectrolytes (CPEs), which are composed of conjugated backbones and side chains with ionic functional groups (Yang et al., 2012; Chang and Leu, 2013; Jo et al., 2016). They can be synthesized with controlled work function and exhibit excellent hole transport properties as compared to the more usual HTL based on PEDOT:PSS. A series of anionic CPEs formed by the copolymerization of 1,4 -bis(4-sulfonatobutoxy)benzene moiety with different counter monomers of thiophene, bithiophene and terthiophene, have shown higher conductivity of ≈10^−4^ S·cm^−1^ due to their self-doping property (Jo et al., 2016). In addition, their higher work function of 5.21 eV as compared to 4.97 eV of PEDOT:PSS facilitates better hole collection by inducing a higher internal electric field within the device (Jo et al., 2016). In addition, the energy levels of a polymer can be changed by its chemical interactions with other materials, however, such interactions may be unique and limited to certain applications in other areas, rather than in the polymer solar cells (Gusain et al., 2017).

Introduction of hybrid organic–inorganic interlayers like TiO_2_/polyethylenimine (PEI) and ZnO/poly[(9,9-bis(30-(N,N-dimethylamino)propyl)-2,7-fluorene)-alt-2,7-(9,9-dioctylfluorene)] (PFN) films acting as ETLs have shown improved electron collection at the electrodes of the inverted solar cell devices (Yang et al., 2014; Lee et al., 2016). A combination of TiO_2_/PEI films has a reduced work function of 4.20 eV as compared to 4.42 eV of the titanium oxide films, yielding a lower energy barrier for electron transport across the cathode/active layer interface and improving electron collection by the electrode (Yang et al., 2014). In addition, such TiO_2_/PEI films also have higher transparency, with reduced series resistance and provide higher electron mobility to the device (Yang et al., 2014). The improvement in the efficiency from 7.38% up to 8.7% has been reported with the application these materials (Yang et al., 2014). Besides TiO_2_, ZnO in combination with other organic materials as a buffer layer has been extensively used to boost the device performance (Chang and Leu, 2013; Kyaw et al., 2013; Trost et al., 2013). The application of ZnO–poly(vinyl pyrrolidone) (PVP) composite sol–gel film as the electron transport layer has been reported to improve the maximum device efficiency from 7.3% up to 8.1% (Small et al., 2012). Further, the application of the non-conjugated polyethylenimine ethoxylated (PEIE) as the polyelectrolyte and a ZnO bilayer as the electron transport layer in the PTB7:PC_71_BM solar cells with an improvement in the efficiency from 7.41% up to 8.38% has also been reported (Jin et al., 2016). As another example, two CPEs, PFN and poly[3-(6-trimethylammoniumhexyl)thiophene] (PTMAHT), were used along with ZnO to form different interlayers (ZnO only, ZnO/PFN and ZnO/PTMAHT) to improve the charge transport across the interfaces (Chang and Leu, 2013). Addition of CPEs to the ZnO not only provides an effective electron-transporting layer but also an excellent surface-smoothening material for ZnO planarization. The role of CPEs is similar to that of PEDOT:PSS smoothening ITO surfaces and reducing leakage currents. The study showed an improved efficiency of 8.54% with the PTB7:PCBM solar cells (Chang and Leu, 2013). Another material, a nanolayer of PEI along with an electron-collecting ZnO buffer layer, was used in the PTB7:PCBM solar cells with an improvement in efficiency from 6.99% up to 8.9% (Woo et al., 2014). It should be noted that some of the effects attributed to these hybrid interlayers (such as Fermi level shift) may in fact be due to the generation of interfacial dipoles at the organic-inorganic interface within these interlayers, as will be discussed in more detail in section Interface Dipoles.

The application of carbon based nanomaterials like carbon-dot-supported nanoparticles, graphene, graphene oxide nanoribbons, and carbon nanotubes for introducing changes in the energy barriers in order to improve the charge carrier extraction has also been explored in different studies (Wei et al., 2007; Choi et al., 2013; Liu et al., 2014a; Gusain et al., 2015; Wang et al., 2015). The application of the carbon-dot-supported silver nanoparticles (CD–Ag nanoparticles) in PTB7:PCBM solar cells showed an improvement in the efficiency from 7.53 to 8.31% (Choi et al., 2013). The surface plasmon resonance effect of CD–Ag nanoparticles allows an additional light absorption leading to an enhanced internal quantum efficiency up to 99% of PTB7:PC_71_BM solar cells as compared to the lower internal quantum efficiency of the devices without CD–Ag nanoparticles (Choi et al., 2013). Similarly, the application of double-walled nanotubes both as the energy conversion material as well as transfer path for charge carriers has been reported (Wei et al., 2007). Further, the application of graphene oxide nanoribbons in P3HT:PCBM solar cells have shown an improvement in the efficiency from 2.20 to 4.14% (Wang et al., 2015). Such improvement in the efficiency was attributed to the proper energy level alignment, good solubility and excellent film-forming capability of graphene oxide nanoribbons (Wang et al., 2015).

A very recent investigation (Brenes-Badilla et al., 2018) demonstrated that the photovoltaic performance of an ITO/PEDOT:PSS/P3HT:PCBM/Ca/Al solar cell is deteriorated (showing the S-kink behavior) after the hole transport layer (PEDOT:PSS) is degraded by contact with the environment, due to the formation of a hole barrier at the PEDOT:PSS/P3HT:PCBM interface. However, the original performance could be recovered by bombarding gold ions on the degraded surface of PEDOT:PSS by means of the metal plasma ion implantation technique. Due to the low energy of the gold ion beam, the implanted gold atoms were located within a few nanometers of the surface, forming gold nanoparticles that have a work function close to the HOMO of P3HT, removing the hole extraction barrier and recovering the solar cell performance.

Sometimes, the use of interlayers can be avoided by properly doping the active material to shift its Fermi level so that a better match to the electrode work function can be attained. It has been shown that with a large carrier concentration within the device, of the order of 10^18^ cm^−3^, the doping shifts the HOMO of the p-layer and LUMO of the n-layer close to the Fermi energy level and creates a depletion layer at the blend/metal interface (Olthof et al., 2009). Such alignment of the energy levels improves the charge carrier injection due to efficient tunneling of electrons and holes through the narrow depletion region, in addition to the conductivity increase by several orders of magnitude (Pfeiffer et al., 2003; Olthof et al., 2009). As an example, we cite the investigation of a solar cell with ITO or gold as the electrodes and p-doped zinc–phthalocyanine (ZnPc) as donor material in BHJs (Blochwitz et al., 2001). It was observed that the HOMO and the vacuum levels in ZnPc layers doped with tetrafluoro-tetracyano-quinodimethane (F4-TCNQ) were shifted, with the width of the space charge layer at the interfaces with electrodes decreasing with doping. It was demonstrated that there was no accumulation of the charge dopant molecules at the interface of ZnPc and ITO, and the barrier changes mainly result from variations of the charge transfer between ZnPc and ITO.

The same phenomenon happens with a highly doped HTL in contact with a metal electrode (Olthof et al., 2009). Thus, doping the HTL leads to a decoupling of the organic layers from the metal contacts and therefore the *V*_*bi*_ due to the doped hole and electron transport layers determines the performance of the device, irrespective of the work functions difference between the metal electrodes (Olthof et al., 2009). However, even when the influence of the metal contacts on the performance remains insignificant, the width and the bending direction of the depletion layer are still affected by the metal contacts (Olthof et al., 2009).

### Interface Dipoles

Before discussing the formation of interface dipoles and their effects on energy barriers and therefore on optoelectronic characteristics of organic solar cells, we must clarify what type of dipoles we will discuss here. In the literature, some authors consider the spontaneously formed charge depletion or accumulation layer near an organic/metal or organic/organic interface as an interface dipole (Hiller et al., 1998). Such cases were briefly discussed in section Physical Mechanism and Definitions, and as seen in Figures 6, 7, they indeed represent a net dipolar region in the device, with charges of opposite signs accumulating on each side. However, these accumulation regions in organic semiconductors are usually quite thick with respect to the molecular scale, typically tens of nm (Hiller et al., 1998). Furthermore, they are intrinsically formed at all interfaces involving semiconductors due to Fermi level equilibration. Thus, we prefer to treat them as an integral part of the energy barriers themselves, as mentioned in section Physical Mechanism and Definitions. Therefore, what we will discuss in this section are molecularly thin (~1 nm) dipolar layers introduced (either intentionally or unintentionally) at various interfaces within the organic solar cells. We will first discuss how they can be used to modify the energy barriers present in polymeric solar cells, and thus their electrical characteristics and efficiency, after which we will describe different ways of introducing such dipolar layers and examples of their effect on solar cell performance.

#### Effect of Interface Dipole on Energy Barriers

The formation of the interface dipoles leaves a significant influence on the device performance which has been explained through various studies carried out on wide variety of organic materials (Collins et al., 1994; Kaspar et al., 1994; Schmidt et al., 1995a,b; Van Slyke et al., 1996; Katz, 1997; Hiller et al., 1998; Lee et al., 1998, 1999; Chen et al., 2013; Liu C. et al., 2015; Kim et al., 2016a,b; Liu et al., 2016; Zhang et al., 2016; Yang et al., 2017; Yu et al., 2017). It has been reported in these studies that interface dipoles strongly influence the process of charge carrier injection/extraction across the electrode interfaces, which in certain cases can lead to the generation of the S-kink in the current-voltage characteristics of the solar cells. On the other hand, the formation of the favorable interface dipoles may improve the charge extraction efficiency by minimization of the interfacial energy barriers, therefore increasing the built in potential, and suppressing charge carrier recombination at the interfaces (Chen et al., 2013; Liu C. et al., 2015; Kim et al., 2016a,b; Liu et al., 2016; Zhang et al., 2016; Yang et al., 2017; Yu et al., 2017). Furthermore, the formation of such surface dipoles is also important in organic heterojunctions, which play important role in photovoltage formation and exciton dissociation occurring at this heterojunction interface (Schmidt et al., 1995a,b; Katz, 1997; Hiller et al., 1998). However, this role of interface dipoles is more prominent for LEDs rather than for the solar cells. It has been suggested that the HOMO/LUMO offsets at these organic heterojunctions can be controlled by interfacial dipoles, thus affecting the efficiencies of organic LED devices (Van Slyke et al., 1996).

**Figure 10:**
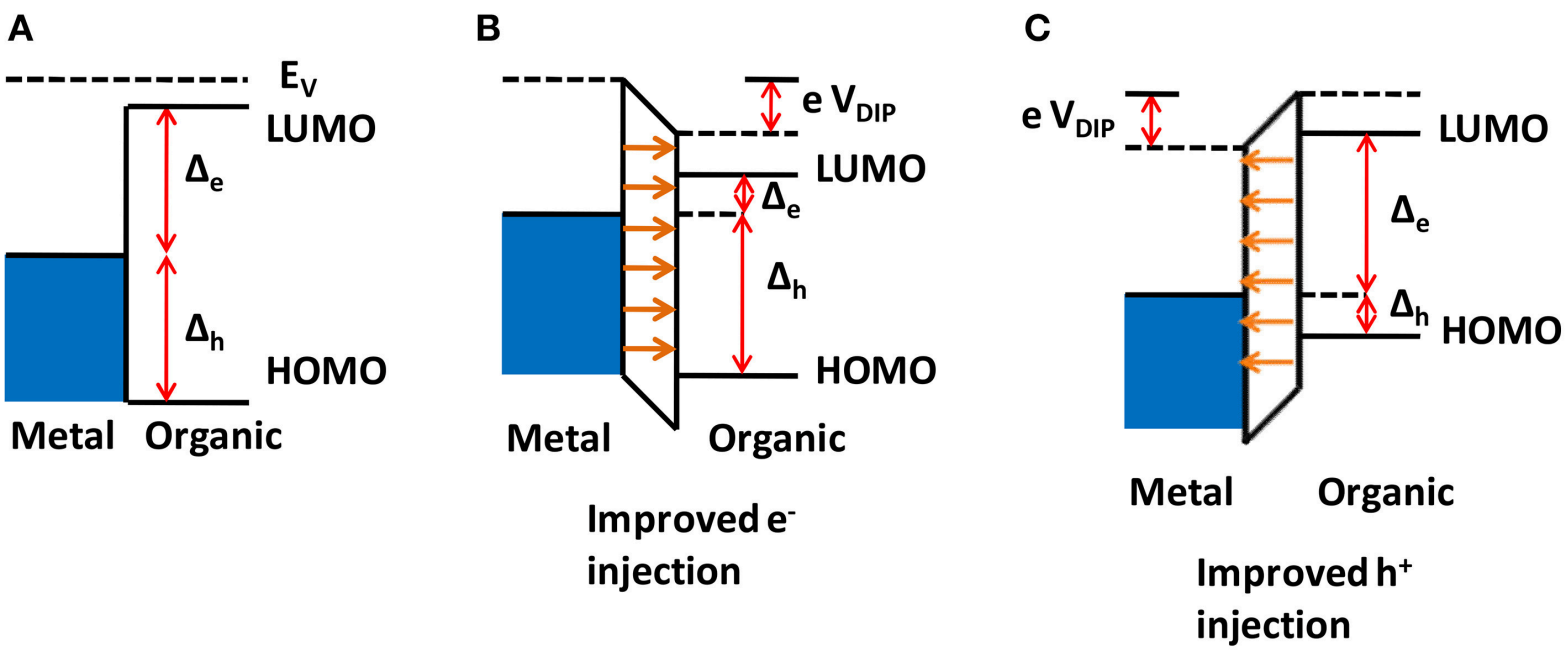
The influence of the formation of the interface dipole on the electronic levels at the metal/organic interface. For simplicity, band bending is neglected. Metal/organic interface **(A)** without a dipole layer, **(B)** with an interfacial dipole pointing away from the metal and **(C)** with an interfacial dipole pointing toward the metal.

The role of the interface dipoles in affecting the charge carrier injection/extraction across the interfaces can be understood from a simple physical picture. As an example, let us consider a metal/organic contact with a layer of dipoles at the interface (Figure 10). For a plane of dipoles whose density per unit area of the dipole component perpendicular to the plane is *P*, the change in electric potential across the dipole layer is *V*_*dip*_ = *P/*ε_0_. This potential difference shifts the energy levels on one side of the dipole layer by Δ*E* = *eV*_*dip*_, where *e* is the elementary charge. This is commonly referred as an effective change in the metal work function by Δ*E*, and therefore affects the energy barriers for charge extraction/injection in the device. As can be seen in Figure 10C, the dipole orientation toward the metal results in the increase of the metal work function and lowering its Fermi level toward the HOMO of the organic layer. As a result, the hole injection barrier (Δ_h_) across the interface is reduced. Similarly, the change in the direction of the interface dipole results in the decrease of the metal work function and a corresponding decrease in the electron injection barrier (Δ_e_), as shown in Figure 10B (Crispin et al., 2002).

#### Physical/Chemical Processes for Interface Dipole Formation

Here we describe the general physical/chemical processes that lead to the formation of the interface dipoles at the interface between a metal and the organic material of the BHJs (Bruening et al., 1997; Blochwitz et al., 2001; Bruner et al., 2002; Crispin et al., 2002; Vazquez et al., 2007). It has been explained that this interface dipole formation occurs from two types of contributions (Crispin et al., 2002). Firstly, there is a partial charge transfer across the interfaces between the metal and the organic layers which results from the chemisorption of the organic molecules on the surface of the metal layer. This partial charge transfer then results in the formation of chemical dipoles across the metal/organic interface. Secondly, surface dipolar layers may also result from physisorption of polar organic molecules oriented at the interface, if they align themselves with a net dipole component perpendicular to the interface. The adsorbed organic molecules can result in the modification of the metal electron density, which further results in the change in the metal surface dipole. For example, the compression of the metal electronic tail due to the presence of adsorbed molecules may also give rise to a potential drop at the interface. This charge rearrangement is termed the “pillow” effect (Vazquez et al., 2007).

However, apart from the understanding of the physical phenomena for the formation of the interface dipoles at the metal/semiconductor interfaces, the fabrication conditions under which these dipoles are formed are also important to be considered. Dipole formation at the metal/organic interfaces of BHJs has been reported to occur during their fabrication due to the uncontrolled effects of factors like exposure of the constituent materials to the atmosphere (Kumar et al., 2009). For example, it has been reported that for BHJ devices using PEDOT (with work function of −5.3 eV) along with BCP as the HTL, there was shifting of the Fermi level from −5.3 to −3.6 eV (Kumar et al., 2009). This shift was introduced by the exposure of BCP to the atmosphere during the solar cell fabrication, and it was attributed to the formation of interface dipoles between the interface between metal-like PEDOT and organic BCP.

In addition, the formation of interface dipoles is not only restricted to the uncontrolled exposure of the materials of the BHJs to the atmosphere, but can also occur during certain chemical modification schemes which are employed to control the effective work function of conductors, including metals, metal oxides and other semiconductor surfaces. This control of the work function is done in order to change energetic barriers for improving charge injection across the interfaces of the materials used in devices like BHJs, LEDs, metal-molecule-metal junctions, metal or semiconductor surfaces (Campbell et al., 1996, 1997; Vilan et al., 2000; Wold and Frisbie, 2001; Ashkenasy et al., 2002; Beebe et al., 2002; Chabinyc et al., 2002; Crispin et al., 2002). One widely used approach reported in these studies is the use of Self-Assembled Monolayers (SAMs) on metal or semiconductor surfaces for controlling the surface composition by the deposition of a closely packed monolayer of molecules with variable terminal group, chain length and film thickness. It is well-known that when a gold surface is exposed to alkanethiol solutions for long times, it leads to the formation of well-organized compact monolayers (Alloway et al., 2003). Such modification of the gold surface leads to the formation of significant interface dipoles, which can be detected by the considerable changes in the work function with the increase of alkyl length chain and fraction of the fluorinated methylene groups in a constant length alkyl chain.

#### Addressing the Performance Issues With Interface Dipoles

Several studies have shown the application of different methods for the formation of favorable interface dipoles in order to improve the device performance. In the case of polymer solar cells based on a ternary blend of PTB7, PC_71_BM, and ICBA, a two-layer structure of zinc oxide (ZnO) and Al-doped zinc oxide (AZO) nanoparticles is used to improve electron extraction (Yang et al., 2017). The study has shown that with the lower work function of AZO as compared to that of ZnO, the built in potential across the organic heterojunction is higher and thus results in more efficient photocurrent extraction and larger open circuit voltages. In addition, with an electron transporting unit of ZnO/AZO/PFN, it has been reported that PFN introduces an interface dipole between the organic active layer and AZO, which leads to an improvement of the device efficiency from 8.34 to 9.17% (Yang et al., 2017).

Some studies have reported the use of SAMs with TiO_2_ or ZnO in order to improve the charge transport across the organic-inorganic interfaces (Hau et al., 2008, 2010). The SAMs have been reported to alter the interfacial properties of different oxides and metallic surfaces. They have also been shown to improve adhesion, compatibility and charge transfer properties at the interface and thereby, reducing back charge recombination (Hau et al., 2008). Further, they have also been used to control the upper layer growth mode and distribution of phases, for the passivation of inorganic surface trap states and shifting the interfacial energy offset between donor–acceptor materials (Hau et al., 2008).

As another example, the application of materials like CPEs in the device has been shown to create favorable dipoles at the electrode/active layer interface which are important for the minimization of the interfacial energy barrier in devices. A series of poly [(9,9-bis(3′-(N,N-dimethylamino)propyl)-2,7-fluorene)-co-2,7-(9,9-dioctylfluorene)] derivatives (PFNs) (PFN_30_, PFN_50_, PFN_70_, and PFN_100_) with different mole ratio of polar groups (–N(C_2_H_5_)_2_) have been used to study the effect of these polar groups on the effective interfacial dipole (Liu et al., 2016). The study reported that with change in the fraction of polar groups, an improvement in the device efficiency from 2.31% for PFN_30_ up to 3.27% for PFN_100_ was achieved (Liu et al., 2016). Another work used a cathode interlayer of fluorene-based CPEs poly[(9,9-bis(6′-(N,N,N-trimethylammonium)hexyl)-2,7-fluorene)-alt-2,7-(9,9-dioctylfluorene)] (PFNBr) and poly[9,9-bis(4′-sulfonatobutyl)fluorene-alt-2,7-(9,9-dioctylfluorene)] (PFSO_3_Na) to improve the device efficiency from 1.28% up to 2.31% and 1.74% up to 3.16%, respectively (Chen et al., 2013). They have shown that the polyelectrolyte can significantly reduce the work function of Al by accumulation of the polar groups at the PFSO_3_Na/Al interface, inducing a favorable interfacial dipole and thus leading to a better energy alignment for electron extraction through the active layer/Al interface (Chen et al., 2013). Applying the ionic liquid tetrabutylphosphonium tetrafluoroborate (P-BF_4_) as cathode interfacial layer in PBDTTT-C:PC_71_BM and PTB7-Th:PC_71_BM solar cells has been shown to improve the device efficiency from 2.76% up to 7.29% and 8.67%, respectively (Yu et al., 2017). It was reported that such improvement results from reduction of the energy barrier due to the formation of an interfacial dipole at the cathode (Yu et al., 2017). Further, the application of liquid-crystal-conjugated polyelectrolytes (LCCPEs) poly[9,9-bis[6-(4-cyanobiphenyloxy)-hexyl]–fluorene–alt-9,9-bis(6-(N,N-diethylamino)-hexyl)-fluorene] (PF6Ncbp) and poly[9,9-bis[6-(4-cyanobiphenyloxy)-hexyl]–fluorene–alt-9,9-bis(6-(N-methylimidazole)-hexyl]-fluorene] (PF6lmicbp) with ZnO interlayer has been reported to improve the device efficiency from 6.4% up to 7.2% and 7.6%, respectively (Liu C. et al., 2015). The study has reported that the spontaneous orientation of liquid-crystal polar groups can induce a dipole moment at the interface which leads to better energy-level alignment (Liu C. et al., 2015). A small-molecule electrolyte, 1,1′-bis(4-hydroxypropyl)-[4,4′-bipyridine]-1,1′-diium bromide (V-OH), used as an interlayer between ZnO and active layer of PTB7:PC_71_BM has been used as a cathode buffer layer for inverted polymer solar cells (Kim et al., 2016b). The molecular structure of the electrolyte has been reported to be responsible for generating a favorable interface dipole which leads to an improvement in the device efficiency from 7.41 to 9.13% (Kim et al., 2016b). Other types of small-molecule electrolytes have also been shown to improve the device efficiency from 7.35% up to 9.20% (Kim et al., 2016a).

Finally, the application of a poly(tetrafluoroethylene) (PTFE) and molybdenum trioxide (MoO_3_) bilayer between ITO and the active layer of PCDTBT:PC_71_BM solar cells has been reported as leading to the formation of an interfacial dipole that increases the surface work function of the ITO anode, which contributes to the extraction of holes and the suppression of carrier recombination at the interface (Zhang et al., 2016). The study has shown a remarkable improvement in the device efficiency from 3.21% up to 7.31% (Zhang et al., 2016).

## Conclusion and Perspectives

The review presents a comprehensive description of the different physical processes occurring at interfaces within organic solar cells (with emphasis on polymer bulk heterojunction devices) that significantly affect their efficiency. The discussion was divided in sections dealing with (i) charge photogeneration and recombination at the donor/acceptor interface, (ii) role of interfacial morphology (vertical phase segregation) on device properties, (iii) effect of charge injection and extraction barriers on the device performance and (iv) methods used for addressing the performance issues brought by energy barriers within the solar cells. We hope that this review has pointed out the extensive amount of research aimed at understanding and controlling these interfacial phenomena on a wide variety of materials. Among the issues discussed here, we highlight a few that we believe are more relevant or should be further investigated. For example, charge recombination is quite detrimental to the overall solar cell performance by reducing the short-circuit current and fill factor, and it is mostly linked to bulk heterojunction morphology and carrier mobility. Thus, a more systematic and quantitative study of this problem is important (Araújo et al., 2019). Although the investigation of interfacial morphology and vertical phase segregation is relatively difficult, requiring advanced characterization techniques, it has an important impact on the understanding of device underperformance, so it needs to be further extended. Another important issue is the use of dipolar layers for energy level alignment. While its physical mechanism is quite well-understood, it is very difficult to have a good characterization of the molecular alignment of these dipoles at the interface in an operating device configuration. Therefore, this is a major challenge that remains to be addressed. It should have also become clear that all these different processes are interrelated to each other in the organic solar cells, and they play a significant role in determining their efficiency. For example, vertical phase segregation may create interfacial dipoles that affect the energy barriers, which in turn may cause charge accumulation within the active layer, leading to increased carrier recombination.

However, a full understanding of all these interfacial issues is very challenging, both due to the complexity and variety of the molecular systems, and to the difficulty of experimentally probing the various types of buried interfaces in these devices (metal/organic, organic/organic, inorganic/organic), especially with techniques that provide a molecular understanding of the interfacial structure and energetics. In this respect, it would be very useful if computer simulations at the electronic/molecular level for these interfaces could become more popular and realistic.

Recently there has been a lot of effort into moving from lab-scale fabrication of these devices to high-throughput production technology for their commercialization (Søndergaard et al., 2011, 2012). Another appealing direction is the high-throughput fabrication of flexible devices through roll-to-roll printing technology (Søndergaard et al., 2011, 2012). These new fabrication technologies bring in important differences to the lab-scale fabrication conditions that use spin-coating and high vacuum thermal evaporation, which are incompatible with high throughput processes. Such translation from lab-scale solution processing deposition techniques to large-scale roll-to-roll methods typically leads to reduced photovoltaic performance. This underperformance may also stem from changes in the relevant interfacial issues due to the unique conditions of these high-throughput technologies. Therefore, a thorough investigation of interfacial issues arising from these new fabrication methods is crucial for their further development.

It should also be observed that the different studies on these interfacial phenomena have ultimately led to the development of new materials in order to address the performance issues. Perspectives in this direction include the design of new self-assembling materials for interface engineering, alternatives to (random) bulk heterojunction using self-assembling conjugated block copolymer structures (de Cuendias et al., 2010), and the development of new active materials, such as solution processable small molecules (Gao et al., 2015), including non-fullerene acceptors (Zhao et al., 2017; Hou et al., 2018). Even with the novel changes in the architecture of the solar cells and use of different processing techniques, the performance of the solar cells is bound to saturate after exploring many processing parameters and conditions. It is with the ingenious introduction of new materials, together with the incorporation of novel techniques, that a brighter future for these solar cells will come.

## Author Contributions

All authors listed have made a substantial, direct and intellectual contribution to the work, and approved it for publication.

### Conflict of Interest Statement

The authors declare that the research was conducted in the absence of any commercial or financial relationships that could be construed as a potential conflict of interest.
